# Genomic inbreeding coefficients using imputed genotypes: assessing differences among SNP panels in Holstein-Friesian dairy cows

**DOI:** 10.3389/fvets.2023.1142476

**Published:** 2023-04-28

**Authors:** Christos Dadousis, Michela Ablondi, Claudio Cipolat-Gotet, Jan-Thijs van Kaam, Raffaella Finocchiaro, Maurizio Marusi, Martino Cassandro, Alberto Sabbioni, Andrea Summer

**Affiliations:** ^1^Department of Veterinary Science, University of Parma, Parma, Italy; ^2^Associazione Nazionale Allevatori della Razza Frisona Bruna e Jersey Italiana (ANAFIBJ), Cremona, Italy; ^3^Department of Agronomy, Food, Natural Resources, Animals, and Environment, University of Padova, Legnaro, Italy

**Keywords:** inbreeding, single nucleotide polymorphism (SNP), imputation, genomics, dairy cattle

## Abstract

The objective of this study was to evaluate the effect of imputation of single nucleotide polymorphisms (SNP) on the estimation of genomic inbreeding coefficients. Imputed genotypes of 68,127 Italian Holstein dairy cows were analyzed. Cows were initially genotyped with two high density (HD) SNP panels, namely the Illumina Infinium BovineHD BeadChip (678 cows; 777,962 SNP) and the Genomic Profiler HD-150K (641 cows; 139,914 SNP), and four medium density (MD): GeneSeek Genomic Profiler 3 (10,679 cows; 26,151 SNP), GeneSeek Genomic Profiler 4 (33,394 cows; 30,113 SNP), GeneSeek MD (12,030 cows; 47,850 SNP) and the Labogena MD (10,705 cows; 41,911 SNP). After imputation, all cows had genomic information on 84,445 SNP. Seven genomic inbreeding estimators were tested: (i) four PLINK v1.9 estimators (F, F_hat1,2,3_), (ii) two genomic relationship matrix (grm) estimators [VanRaden's 1^st^ method, but with observed allele frequencies (F_grm_) and VanRaden's 3^rd^ method that is allelic free and pedigree dependent (F_grm2_)], and (iii) a runs of homozygosity (roh) – based estimator (F_roh_). Genomic inbreeding coefficients of each SNP panel were compared with genomic inbreeding coefficients derived from the 84,445 imputation SNP. Coefficients of the HD SNP panels were consistent between genotyped-imputed SNP (Pearson correlations ~99%), while variability across SNP panels and estimators was observed in the MD SNP panels, with Labogena MD providing, on average, more consistent estimates. The robustness of Labogena MD, can be partly explained by the fact that 97.85% of the SNP of this panel is included in the 84,445 SNP selected by ANAFIBJ for routine genomic imputations, while this percentage for the other MD SNP panels varied between 55 and 60%. Runs of homozygosity was the most robust estimator. Genomic inbreeding estimates using imputation SNP are influenced by the SNP number of the SNP panel that are included in the imputed SNP, and performance of genomic inbreeding estimators depends on the imputation.

## 1. Introduction

The evolution in recent years of genotyping technologies enabled a continuous drop in costs and increased availability in the market of various single nucleotide polymorphism (SNP) microarrays (hereafter denoted as SNP panels) for livestock species diverse in quantity (number of SNP) and quality (e.g., SNP targeting specific genes). This promoted advanced genomic tools in animal breeding, but also led many breeding companies to genotype, in time, different groups of animals with diverse SNP panels. Moreover, the combination of overlapping SNP among panels and imputation pipelines allows to further reduce costs. Nowadays, it is a common practice to genotype few core animals with high density (HD) SNP panels (or whole genome sequencing), genotype a high number of animals with low density or medium density (LD and MD, respectively) SNP panels and to impute the LD/MD to HD genotypes, hence ending up with a common number of imputed genotypes for all genotyped animals (1, 2). Very analogous is the imputation of low coverage sequencing data to high coverage (3). The imputation SNP data can be used for genomic predictions, genome-wide association analyses, genetic diversity studies within and across populations, etc. Moreover, imputation SNP data can be used for estimating genetic relationships among animals and inbreeding coefficients (termed as genomic inbreeding), that were traditionally estimated via pedigree data. For the latter, there are various factors that can influence genomic inbreeding coefficients, such as methodology (e.g., summing homozygosity over individual SNP vs. summing homozygous blocks), associated parameters within each estimator (e.g., parameters to define a homozygous block), SNP quality control, imputation method, etc. (4–6).

The imputation procedures in livestock breeding programs increased the interest to assess the effect of SNP panels in estimating inbreeding coefficients. The objective of this study was to evaluate the effect of imputation of SNP on the estimation of genomic inbreeding coefficients. Thus, we extended a previous work on genomic inbreeding with imputed SNP (6), aiming to quantify differences among SNP panels that cows were genotyped with (i.e., MD vs. HD). Two HD (Illumina Infinium BovineHD BeadChip and Genomic Profiler HD-150K) and four MD (GeneSeek Genomic Profiler 3, GeneSeek Genomic Profiler 4, GeneSeek Genomic MD and Labogena MD) SNP panels were analyzed. Genomic inbreeding coefficients were estimated with seven commonly used estimators. Comparisons between genotyped – imputation inbreeding coefficients were made for each SNP panel and estimator.

## 2. Materials and methods

### 2.1. Animals and genotypes

**Figure 1:**
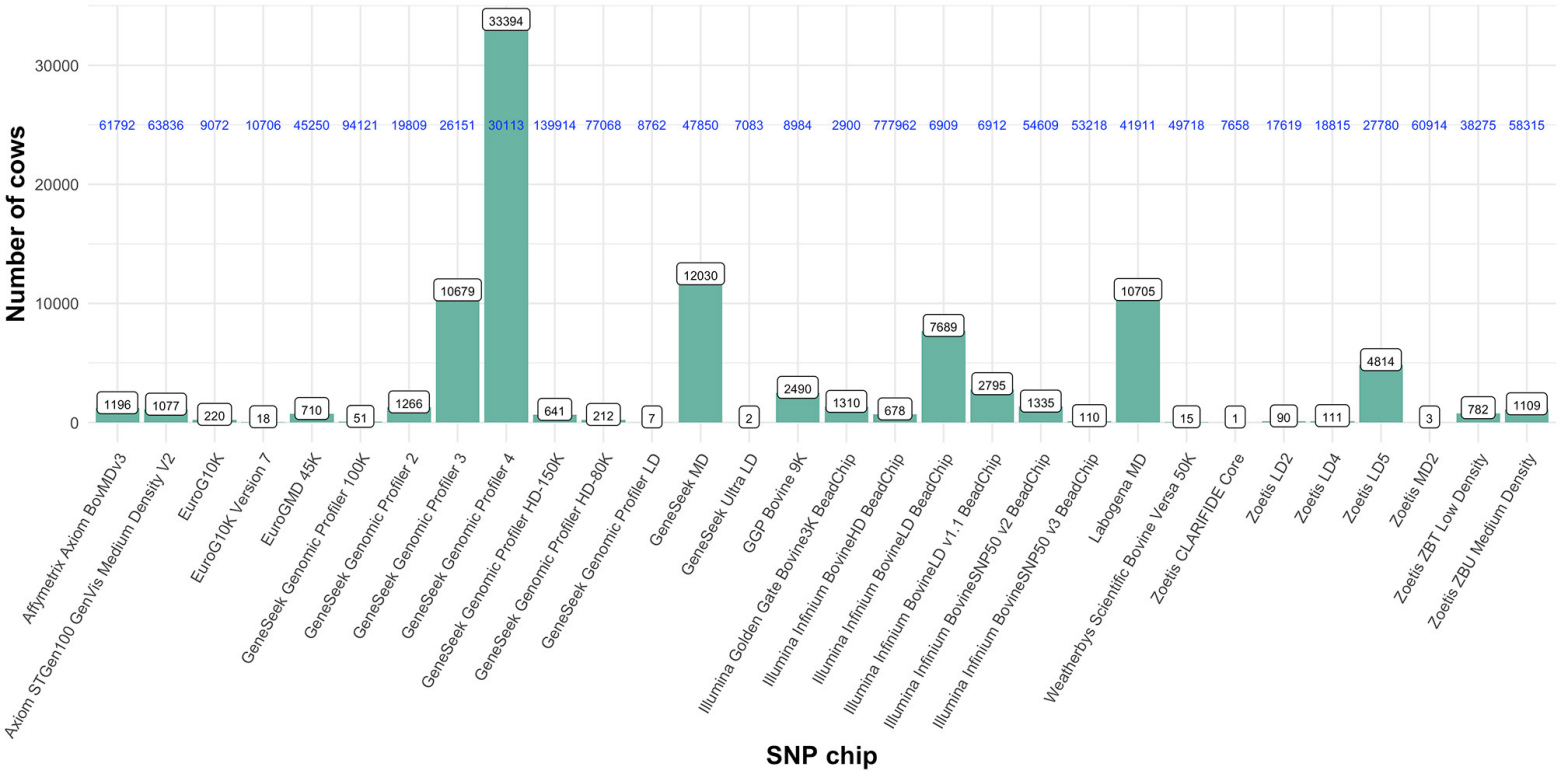
Cows and genotype panels available from which were selected two high density SNP panels (Illumina Infinium BovineHD BeadChip and GeneSeek Genomic Profiler HD-150K) and four medium density SNP panels (GeneSeek Genomic Profiler 3, GeneSeekGenomic Profiler 4, GeneSeek MD and Labogena MD). Numbers on the top of the bars show the number of cows genotyped within each SNP panel. Horizontal numbers in blue show the total number of SNP included in each SNP panel.

The available dataset contained 95,540 Italian Holstein dairy cows, all registered to the official herd book of the Italian National Association of Holstein, Brown and Jersey Breeders (ANAFIBJ). Cows were born between 1998 and 2020 and genotyped with 30 different SNP panels of varying densities (from 3k to 777k; Figure 1). From those, we selected 68,127 Italian Holstein dairy cows genotyped with two high density (HD) SNP panels, namely the Illumina Infinium BovineHD BeadChip (678 cows; 777,962 SNP) and the Genomic Profiler HD-150K (641 cows; 139,914 SNP), and four medium density (MD) SNP panels: GeneSeek Genomic Profiler 3 (10,679 cows; 26,151 SNP), GeneSeek Genomic Profiler 4 (33,394 cows; 30,113 SNP), GeneSeek MD (12,030 cows; 47,850 SNP) and the Labogena MD (10,705 cows; 41,911 SNP). A dataset of 84,445 common SNP (on the 29 autosomes) was created. Those SNP are pre-selected and used in the routine genomic evaluations of ANAFIBJ. Cows genotyped with the four MD SNP panels were imputed, while those with the two HD SNP panels were degraded to the 84,445 common SNP. The imputation was carried out in an improved version of the PedImpute software (7) for faster computations. After imputation, SNP quality control included: (i) call rate < 95%, (ii) parent-offspring SNP mismatch > 0.01, (iii) minor allele and genotype (< 0.02 and < 0.001, respectively) frequencies and (iv) extreme deviation from Hardy–Weinberg equilibrium (*P* < 0.005).

### 2.2. Inbreeding coefficients

In scientific literature, inbreeding coefficient is abbreviated either as F or f. To be consistent with software abbreviation, we used F to denote the first genomic inbreeding estimator of PLINK v.1.9 software [(8); http://pngu.mgh.harvard.edu/purcell/plink/]. We adopted “*f* ” to denote inbreeding coefficient and *f*_*SNP*_ for referring to whole genome SNP based inbreeding coefficients. Seven genomic inbreeding estimators were tested, and genomic inbreeding coefficients were calculated for the 68,127 cows for each estimator:

- Four estimators (F, F_hat1,2,3_) implemented in PLINK v1.9 [(8); http://pngu.mgh.harvard.edu/purcell/plink/]. F (flag –*het* in PLINK v1.9) was proposed by Li and Horvitz (1953) and counts the proportion of homozygous SNP. F_hat1 − 3_ were primarily implemented in the GCTA software (9, 10) and can be obtained simultaneously with the flag –*ibc* in PLINK v1.9. More precisely, F_hat1_ is estimated as $\frac{1}{n}\overset{n}{\underset{m=1}{\sum }}\frac{{\left({\text{X}}_{m}-2p_{m}\right)}^{2}}{2p_{m}q_{m}}-1$, where **X** is the genotype matrix based on the number of copies of the defined reference allele, with p and q being the frequencies of the reference and alternative alleles, respectively and *m* is the number of SNP. F_hat2_ measures the excess of homozygosity ($1-\frac{1}{n}\overset{n}{\underset{m=1}{\sum }}\frac{{\text{X}}_{m}\left(2-{\text{X}}_{m}\right)}{2p_{m}q_{m}}$) and differs from F in the sense that F_hat2_ is a sum of ratios, while F is a ratio of sums (11). F_hat3_ is estimated as $\frac{1}{n}\overset{n}{\underset{m=1}{\sum }}\frac{{\text{X}}_{m}^{2}-\left(1\;+\;2p_{m}\right){\text{X}}_{m}+2p_{m}^{2}}{2p_{m}q_{m}}$ and reflects the inbreeding definition of Wright stated as correlation between uniting gametes (12, 13).
- Two genomic relationship matrix (grm) – based inbreeding estimators (based on VanRaden's 1^st^ and 3^rd^ methods). The first method (F_grm_) (14– 16) was estimated as follows: grm = $\frac{{\text{Z}\text{Z}}^{\prime }}{2\sum q_{m}\left(1-q_{m}\right)}$, where **Z** = **X** – 2(q_m_ – 0.5); where F_grm_ = *diag*(grm) – 1. To alleviate the problem of using observed allele frequencies in F_grm_, a simplified version of VanRaden's 3^rd^ proposed method was used F_grm2_, where we regressed the diagonal of **XX**′ on pedigree inbreeding coefficients (F_ped_) to get the mean and the slope and then to obtain $F_{grm2}=\;\frac{diag(XX^{\prime })-mean}{slope}-mean$, where mean and slope are the estimates of the previous regression. Both estimators were determined without considering a base population or AI sire information to calibrate the diagonal elements of **XX**′, as, for e.g., reported by (17),
- A roh-based estimator (F_roh_), where F_roh_ expresses the sum of rohs identified in an individual to the total genome length. We used the consecutive runs method in the R software (*v. 3.6.3*) package *detectRUNS v. 0.9.5* (18–20). To define a roh we set the minimum length of roh to 1 Mbp and a minimum of 15 SNPs/ROH. Moreover, we allowed one heterozygous SNP within a roh to account for possible genotyping errors. In a previous study (6), we focused on the differences among genomic inbreeding estimators. In that study, two more genomic inbreeding estimators were included that were simplifications of the F and F_roh_ estimators, namely F_PH_ and F_ROH2_, respectively, as reported in (6). Due to their high correlations, we decided to exclude those estimators from the current study. Moreover, the grm-based estimator (F_GRM05_; with allele frequencies set to 0.5) described in (6) was highly correlated with F and was not considered in the current analysis.

In addition, pedigree based inbreeding coefficients (F_ped_) were also estimated in the *pedigree* R package (18, 21). This estimation does not consider genetic groups and assigns the value of 0 for missing ancestors. Pedigree consisted of 393,607 cattle with 10 generations depth with a pedigree completeness index (22), estimated in R package *optiSel* (23), of 0.99.

Pairwise comparisons were made between genotyped-imputation *f*_*SNP*_ for each SNP panel and genomic inbreeding estimator. In each panel, only those genotyped SNP included in the preselected imputation set of 84,445 SNP were considered (because the rest of the SNP were automatically omitted from the imputation pipeline of ANAFIBJ). This means that the genotyped SNP per panel were 79,900 (Illumina Infinium BovineHD BeadChip), 77,085 (Genomic Profiler HD-150K), 13,870 (GeneSeek Genomic Profiler 3), 16,862 (GeneSeek Genomic Profiler 4), 27,331 (GeneSeek MD) and 40,218 (Labogena MD) (Table 1). Average SNP distance per chromosome for each panel and the imputation data was estimated. Results were also summarized over genomic inbreeding estimators across the different SNP panels. Pearson and Spearman correlations were considered for estimating the consistency of inbreeding coefficients between genotyped – imputation SNP for each SNP panel. The imputation data of 84,445 SNP was a mixture of genotyped and imputed SNP, hence the term imputation SNP was adopted herein rather imputed SNP.

**Table 1:** Number of single nucleotide polymorphisms per chromosome in the imputed dataset and each genotype panel.

**Chr**	**Imputation**	**Illumina Infinium BovineHD BeadChip**	**GeneSeek Genomic Profiler HD-150K**	**GeneSeek Genomic Profiler 3**	**GeneSeek Genomic Profiler 4**	**GeneSeek MD**	**Labogena MD**
1	5,255	4,980	4,750	794	981	1,673	2,628
2	4,398	4,150	4,019	627	770	1,506	2,142
3	4,120	3,868	3,736	661	833	1,408	2,040
4	3,938	3,748	3,561	523	643	1,238	1,949
5	3,835	3,642	3,506	820	976	1,298	1,674
6	4,004	3,792	3,649	590	759	1,227	1,960
7	3,617	3,447	3,291	569	673	1,102	1,711
8	3,794	3,575	3,428	538	670	1,179	1,853
9	3,431	3,261	3,140	526	670	1,189	1,649
10	3,436	3,250	3,155	479	582	1,111	1,685
11	3,642	3,412	3,304	545	667	1,129	1,724
12	2,815	2,660	2,569	441	512	882	1,306
13	2,827	2,680	2,585	437	536	946	1,382
14	2,887	2,760	2,665	497	604	924	1,405
15	2,880	2,709	2,627	507	604	889	1,369
16	2,770	2,609	2,515	443	543	904	1,320
17	2,553	2,416	2,350	370	449	792	1,223
18	2,352	2,226	2,171	536	642	784	1,012
19	2,340	2,216	2,138	539	641	812	1,071
20	2,631	2,482	2,357	470	598	924	1,323
21	2,383	2,234	2,151	440	534	797	1,111
22	2,137	2,022	1,964	311	386	682	997
23	1,922	1,825	1,787	410	495	653	881
24	2,124	2,031	1,937	331	394	659	953
25	1,608	1,518	1,500	312	374	518	757
26	1,800	1,704	1,668	301	356	566	827
27	1,605	1,529	1,499	257	296	492	757
28	1,618	1,539	1,478	278	315	516	721
29	1,723	1,615	1,585	318	359	531	788
Total	84,445	79,900	77,085	13,870	16,862	27,331	40,218
Percentage^a^	/	94.62	91.28	16.42	19.97	32.37	47.63

a Percentage of SNP of the SNP panel included in the imputation data.

## 3. Results

### 3.1. Similarities among the SNP panels and the imputed SNP data

Table 1 and Supplementary Figure 1 summarize the number and density, respectively, of SNP per chromosome for each SNP panel and the imputation data. The number of SNP included in the preselected 84,445 imputation SNP varied for each SNP panel from 79,900 (Illumina Infinium BovineHD BeadChip) to 13,870 (GeneSeek Genomic Profiler 3), that is corresponding to 94.6% and 16.4%, respectively.

**Figure 2:**
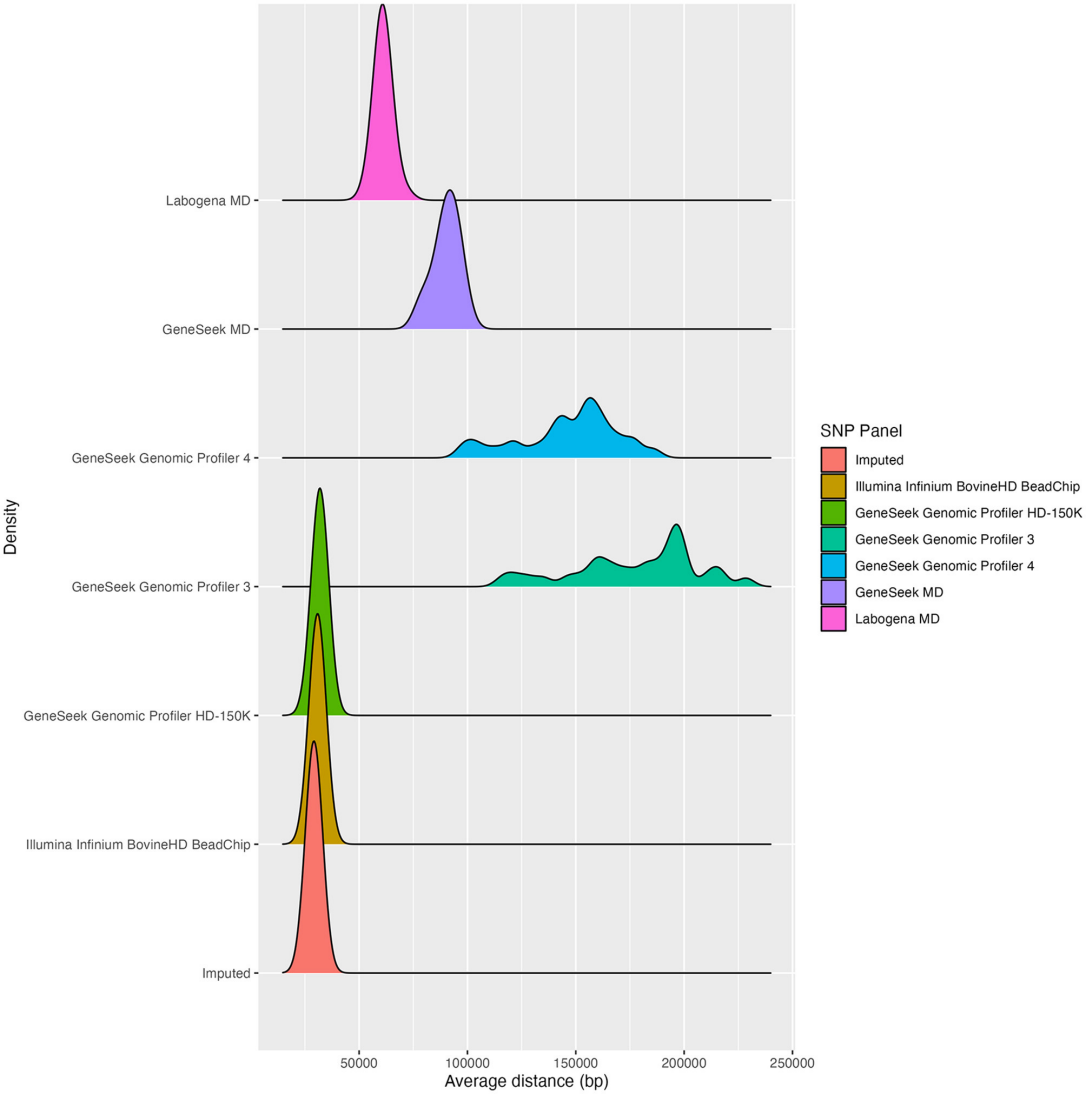
SNP distance distribution over the 29 autosomes for each of the SNP panels.

The average SNP distance over the 29 autosomes (Figure 2) was 29,138 bp for the imputation set, 30,791 bp for the Illumina Infinium BovineHD BeadChip and 31,867 bp for the Genomic Profiler HD-150K. Higher average SNP distances were observed for Labogena MD (61,119 bp), GeneSeek MD (90,200 bp), and the GeneSeek Genomic Profiler 4 and 3 (146,433 and 177,375 bp, respectively). The SNP distance distributions over the 29 autosomes were comparable for the two HD SNP panels and the imputation set. Regarding MD SNP panels, the Labogena MD was more closely to the HD SNP panels, followed by GeneSeek MD, while the GeneSeek Genomic Profiler 4 and GeneSeek Genomic Profiler 3 clearly diverged from the rest, with both SNP panels consisting of a mixture of distributions.

### 3.2. Correlations of inbreeding coefficients between genotyped and imputation SNP

Descriptive statistics of the pedigree and SNP inbreeding coefficients are reported in Table 2. The average F_ped_ was 0.05 for the cows genotyped with Illumina Infinium BovineHD BeadChip, 0.07 for GeneSeek Genomic Profiler 3, 4 and Labogena MD and 0.08 for Genomic Profiler HD-150K and GeneSeek MD. Average *f*_*SNP*_ was close to 0 (for both genotyped and imputed SNP) for the genomic estimators, except for F_grm2_ and F_roh_. Specifically, average *f*_*SNP*_ across all SNP panels for F_grm2_ varied between−0.92 and−0.95 with inbreeding coefficients being always negative. The highest mean *f*_*SNP*_ across all SNP panels was observed for F_roh_ (0.11 to 0.16). Moreover, although the mean *f*_*SNP*_ was, in general, equal between genotyped – imputed SNP for all estimators, the space of the inbreeding coefficients differed when estimated using genotyped vs. imputation SNP. This was observed for all estimators and SNP panels. The most consistent results were found for the two HD SNP panels when *f*_*SNP*_ was estimated with F_roh_. For e.g., F in the group of cows genotyped with the GeneSeek Genomic Profiler 3 varied between −0.35 to 0.26 for the genotyped and−0.16 to 0.82 for the imputation SNP data. Similarly, for the same group of cows, F_roh_ ranged between 0.00–0.37 and 0.02–0.60 for the genotyped and imputation SNP, respectively.

**Table 2:** Mean, standard deviation (superscript) and range (subscript) of the pedigree and the genomic inbreeding coefficients for each genotyping panel.

**Estimator**	**Genomic information**	**Illumina Infinium BovineHD BeadChip**	**GeneSeek Genomic Profiler HD-150K**	**GeneSeek Genomic Profiler 3**	**GeneSeek Genomic Profiler 4**	**GeneSeek MD**	**Labogena MD**
**Pedigree**							
F_ped_		$0.05_{\left[0,0.28\right]}^{0.02}$	$0.08_{\left[0,0.29\right]}^{0.03}$	$0.07_{\left[0,0.31\right]}^{0.02}$	$0.07_{\left[0,0.33\right]}^{0.02}$	$0.08_{\left[0,0.31\right]}^{0.03}$	$0.07_{\left[0,0.31\right]}^{0.02}$
**PLINK** ^a^							
F	Genotyped	${-0.01}_{\left[-0.30,0.24\right]}^{0.04}$	$0.01_{\left[-0.09,0.31\right]}^{0.05}$	${-0.01}_{\left[-0.35,0.26\right]}^{0.04}$	$0.00_{\left[-0.44,0.36\right]}^{0.04}$	$0.00_{\left[-0.60,0.94\right]}^{0.05}$	${-0.01}_{\left[-0.22,0.37\right]}^{0.04}$
	Imputation	${-0.01}_{\left[-0.30,0.24\right]}^{0.04}$	$0.01_{\left[-0.09,0.31\right]}^{0.05}$	$0.00_{\left[-0.16,0.82\right]}^{0.05}$	$0.01_{\left[-0.20,0.78\right]}^{0.05}$	$0.01_{\left[-0.38,0.79\right]}^{0.06}$	$0.0_{\left[-0.17,0.55\right]}^{0.04}$
F_hat1_	Genotyped	${-0.03}_{\left[-0.27,0.27\right]}^{0.07}$	$0.00_{\left[-0.18,0.50\right]}^{0.09}$	${-0.02}_{\left[-0.24,1.81\right]}^{0.07}$	${-0.01}_{\left[-0.29,11.10\right]}^{0.17}$	$0.00_{\left[-0.42,65.74\right]}^{0.61}$	${-0.01}_{\left[-0.21,1.46\right]}^{0.09}$
	Imputation	${-0.03}_{\left[-0.26,0.27\right]}^{0.07}$	${-0.01}_{\left[-0.20,0.52\right]}^{0.10}$	${-0.02}_{\left[-0.21,1.33\right]}^{0.11}$	$0.00_{\left[-0.22,2.28\right]}^{0.12}$	$0.00_{\left[-0.31,31.04\right]}^{0.31}$	${-0.01}_{\left[-0.19,1.84\right]}^{0.10}$
F_hat2_	Genotyped	${-0.01}_{\left[-0.30,0.25\right]}^{0.06}$	$0.01_{\left[-0.45,0.29\right]}^{0.09}$	${-0.01}_{\left[-1.89,0.26\right]}^{0.07}$	$0.00_{\left[-11.11,0.36\right]}^{0.17}$	$0.00_{\left[-3.50,0.30\right]}^{0.10}$	${-0.01}_{\left[-1.32,0.39\right]}^{0.09}$
	Imputation	${-0.01}_{\left[-0.28,0.26\right]}^{0.06}$	$0.01_{\left[-0.48,0.29\right]}^{0.10}$	$0.00_{\left[-1.28,0.82\right]}^{0.09}$	$0.00_{\left[-2.27,0.80\right]}^{0.11}$	$0.01_{\left[-3.66,0.69\right]}^{0.11}$	$0.00_{\left[-1.69,0.50\right]}^{0.10}$
F_hat3_	Genotyped	${-0.01}_{\left[-0.23,0.25\right]}^{0.04}$	$0.01_{\left[-0.08,0.32\right]}^{0.03}$	${-0.01}_{\left[-0.28,0.26\right]}^{0.03}$	$0.00_{\left[-0.35,0.78\right]}^{0.03}$	$0.00_{\left[-0.47,32.86\right]}^{0.30}$	${-0.01}_{\left[-0.18,0.29\right]}^{0.03}$
	Imputation	${-0.01}_{\left[-0.22,0.26\right]}^{0.04}$	$0.01_{\left[-0.08,0.32\right]}^{0.03}$	$0.00_{\left[-0.12,1.05\right]}^{0.05}$	$0.00_{\left[-0.15,1.16\right]}^{0.04}$	$0.01_{\left[-0.27,15.60\right]}^{0.15}$	$0.00_{\left[-0.13,0.86\right]}^{0.04}$
**grm** ^b^							
F_grm_	Genotyped	${-0.01}_{\left[-0.31,0.27\right]}^{0.05}$	$0.01_{\left[-0.13,0.36\right]}^{0.05}$	${-0.01}_{\left[-0.27,0.26\right]}^{0.04}$	${-0.03}_{\left[-1.05,0.44\right]}^{0.16}$	$0.00_{\left[-0.46,1.87\right]}^{0.05}$	${-0.01}_{\left[-0.23,0.28\right]}^{0.04}$
	Imputation	${-0.01}_{\left[-0.32,0.28\right]}^{0.05}$	$0.01_{\left[-0.13,0.37\right]}^{0.05}$	$0.00_{\left[-0.22,0.93\right]}^{0.06}$	${-0.02}_{\left[-1.08,0.91\right]}^{0.16}$	$0.00_{\left[-0.45,0.71\right]}^{0.06}$	$0.00_{\left[-0.26,0.67\right]}^{0.05}$
F_grm2_	Genotyped	${-0.95}_{\left[-1.36,-0.63\right]}^{0.05}$	${-0.92}_{\left[-1.00,-0.70\right]}^{0.04}$	${-0.93}_{\left[-1.26,-0.67\right]}^{0.04}$	${-0.93}_{\left[-1.85,-0.62\right]}^{0.13}$	${-0.92}_{\left[-1.46,-0.19\right]}^{0.04}$	${-0.93}_{\left[-1.13,-0.59\right]}^{0.03}$
	Imputation	${-0.95}_{\left[-1.36,-0.63\right]}^{0.05}$	${-0.92}_{\left[-1.01,-0.70\right]}^{0.04}$	${-0.93}_{\left[-1.21,-0.08\right]}^{0.05}$	${-0.93}_{\left[-1.99,-0.26\right]}^{0.16}$	${-0.92}_{\left[-1.37,-0.41\right]}^{0.05}$	${-0.93}_{\left[-1.27,-0.45\right]}^{0.04}$
**roh** ^c^							
F_roh_	Genotyped	$0.12_{\left[0.02,0.34\right]}^{0.03}$	$0.15_{\left[0.07,0.41\right]}^{0.05}$	$0.11_{\left[0.00,0.37\right]}^{0.03}$	$0.13_{\left[0.00,0.43\right]}^{0.03}$	$0.15_{\left[0.00,0.86\right]}^{0.04}$	$0.16_{\left[0.02,0.47\right]}^{0.03}$
	Imputation	$0.12_{\left[0.02,0.34\right]}^{0.03}$	$0.15_{\left[0.07,0.40\right]}^{0.05}$	$0.14_{\left[0.02,0.60\right]}^{0.04}$	$0.15_{\left[0.03,0.51\right]}^{0.04}$	$0.15_{\left[0.01,0.40\right]}^{0.04}$	$0.16_{\left[0.01,0.47\right]}^{0.03}$

a Genomic inbreeding estimates based on PLINKv1.9 software;

b Genomic inbreeding estimates based on genomic relationship matrices (grm);

c Genomic inbreeding estimates based on runs of homozygosity (roh).

Negative *f*_*SNP*_ were found for all estimators, except F_roh_. Although with pedigree this cannot happen, with SNP data this is possible. Theoretically, inbreeding coefficients below zero reflect potential gain of genetic variability, given an unselected base population consisted of unrelated individuals. The interpretation and the theoretical background of inbreeding coefficients has been elaborated in previous studies (5, 6).

Pairwise comparisons between genotyped vs. imputation *f*_*SNP*_ for each SNP panel were investigated (Supplementary Figure 2); Pearson correlations are reported, except if stated otherwise. For the two HD SNP panels correlations between genotyped-imputation *f*_*SNP*_ were close to one (≥ 0.98) for all genomic inbreeding estimators (Supplementary Figures 2A, B). For the four MD SNP panels, correlations ranged between 0.65 (GeneSeek Genomic Profiler 3; Supplementary Figure 2C) and 0.85 (GeneSeek MD and Labogena MD; Supplementary Figures 2E, F). However, for MD SNP panels correlations between genotyped-imputation *f*_*SNP*_ varied across estimators. More precisely, for the three GeneSeek SNP panels, F_hat2_ had the lowest correlations, ranging between 0.51 (GeneSeek Genomic Profiler 4; Supplementary Figure 2D) to 0.68 (GeneSeek MD; Supplementary Figure 2E). For Labogena MD (Supplementary Figure 2F), F_hat3_ had the lowest correlation (0.77) between genotyped-imputation *f*_*SNP*_. Moreover, for GeneSeek Genomic Profiler 3 the estimators F, F_hat3_ and F_grm2_ had correlations ~0.60, F_hat1_ and F_grm_ values ranged between 0.68-0.75 with F_roh_ being more consistent (~0.79) compared to the rest of the estimators. For GeneSeek Genomic Profiler 4, the lowest correlation (0.55) was observed for F_hat1_, followed by F_hat3_ and F_grm_ (~0.62 for both), F (0.68), F_roh_ (0.89) and F_grm2_ (0.98). For GeneSeek MD (Supplementary Figure 2E), the estimators F, F_grm_ and F_grm2_ had correlations ~0.80 between genotyped-imputation *f*_*SNP*_, while F_hat1_, F_hat3_ and F_roh_ had values close to one (~0.97). In this case, however, it should be noted that an extreme and influential inbreeding coefficient was observed for F_hat1_ and F_hat3_ that impacts the values of Pearson correlations. In the case of Labogena MD (Supplementary Figure 2F), all estimators except F_hat3_ had correlations of between 0.82-0.89 with the highest (0.98) observed for F_roh_; correlations between genotyped – imputed *f*_*SNP*_ for F_hat3_ were at 0.77. Moreover, for Labogena MD the inbreeding coefficients of the imputed SNP had always greater variability and higher values than the genotyped SNP, across all estimators (Table 2). This, in general, was also observed for the three MD GeneSeek estimators. However, for the three MD GeneSeek panels there were cases, especially for F_hat1−3_ were *f*_*SNP*_ estimated from genotyped SNP showed greater variability.

To further investigate the differences between genotyped vs. imputation *f*_*SNP*_, pairwise comparison with F_ped_ were made for all SNP panels and estimators (Supplementary Figure 3). We assume that the most accurate inbreeding coefficients should have higher correlation to F_ped_. In general, the genotyped data had higher correlations to F_ped_ compared to the imputation set. Opposite results were found for F_hat1,2_ for GeneSeek Genomic Profiler 4, where *f*_SNP_ estimated from the imputation SNP were higher correlated to F_ped_ compared to *f*_SNP_ estimated using the only the genotyped SNP. No differences were found for the two HD SNP panels.

**Figure 3:**
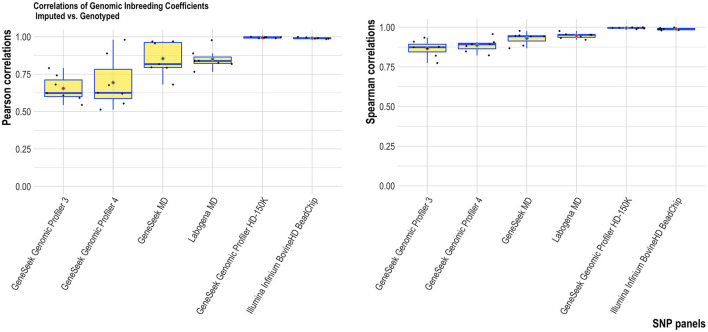
Average Pearson **(left)** and Spearman **(right)** correlations of the genomic inbreeding estimators tested for each SNP panel. Horizontal bars within each boxplot represent the median, and red rhombus the mean.

**Figure 4:**
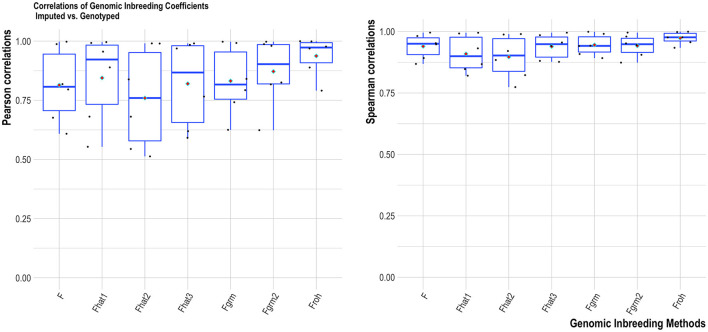
Average Pearson **(left)** and Spearman **(right)** correlations of the tested SNP panels for each genomic inbreeding estimator. Horizontal bars within each boxplot represent the median, and red rhombus the mean.

Overall, the two HD SNP panels had consistent results between genotyped-imputation *f*_*SNP*_ (correlations close to one). For the four MD SNP panels, higher correlations were found for the Labogena MD (summarized over all estimators), followed by the GeneSeek MD, GeneSeek Genomic Profiler 4 and GeneSeek Genomic Profiler 3 (Figure 3). F_roh_ provided the most robust results across all genomic inbreeding estimators tested (Figure 4). Spearman correlations were always higher compared to Pearson correlations (with the former being able to capture monotonic patterns).

## 4. Discussion

Inbreeding coefficients are traditionally estimated from pedigree data and used to characterize diversity, evolution, and population structure. The study of inbreeding can be applied to individual animals, herd, consortium (e.g., dairy chains or semen companies) and population levels (24–26). It is also used in livestock breeding and conservation programs to organize matings and manage the level of relationship among individuals of a given population. Whole genome SNP data allowed the estimation of the realized level of homozygosity of an individual, compared to the expectations derived from pedigree information. However, it is important to keep in mind that homozygosity might be caused by either common ancestors (homozygosity by descent; autozygosity) or by other evolutionary processes. In the latter case, homozygosity represents an identical by state situation termed allozygosity. The two forms of homozygosity practically are not straightforward to be distinguished.

The rationale of the present work was driven by applied methods of estimating genomic inbreeding coefficients with whole genome imputed SNP data, during routine genomic evaluations in dairy cattle breeding programs. Our work is not critical on the SNP panels evaluated herein *per se*, rather on the way they are applied in breeding programs. The rapid increase in number and quality of SNP panels in the market, the drastic drop for genotyping and novel imputation methods resulted in genotyping subgroups within breeding populations. For instance, in the ANAFIBJ genomic breeding program to date, 43 SNP panels have been utilized to genotype different groups of cattle. This situation is representative of other genomic breeding programs mainly in cattle (27, 28), broilers (29) and swine (30, 31).

In the dataset analyzed in the current study, 10,679 cows were genotyped with GeneSeek Genomic Profiler 3 (containing 26,151 SNP), and 33,394 cows with GeneSeek Genomic Profiler 4 (containing 30,113 SNP). However, for those cows only 13,870 and 16,862 SNP were used in the imputation data (representing 16.4 and 20% of the imputation set, respectively). Less SNPs of these chips were selected because they have a lower overlap with other DNA chips. This means, that (i) ~50% of the SNP of those panels are omitted and (ii) cows genotyped with those SNP panels have ~80–85% of their genotypes imputed. For some of those cows discrepancies were found between observed vs. imputed SNP genomic inbreeding coefficients, with the question being which estimates represent the real state. Moreover, results varied among estimators.

To address this question we used as a baseline the F_ped_, assuming that a higher correlation with F_ped_ is favorable. Our results showed that, in general, the genotyped *f*_*SNP*_ were strongly correlated to the F_ped_ for all estimators (Supplementary Figure 3) in the MD SNP panels, compared to *f*_*SNP*_ estimated with SNP from the imputation set. However, variability was observed among estimators on the the actual difference between genotyped – imputation *f*_*SNP*_. For e.g., for the GeneSeek Profilers 3 Pearson correlations between F_ped_ and each of the genomic estimators were 0.56,−0.12, 0.45, 0.36, 0.04, 0.58 and 0.61, while with the imputation SNP correlations were of 0.38,−0.25, 0.45, 0.16, 0.03, 0.38 and 0.48 for F, F_hat1_, F_hat2_, F_hat3_, F_grm_, F_grm2_ and F_roh_, respectively. Moreover, imputation increased the correlations between the pairwise comparison of the genomic inbreeding estimators. For instance, in the GeneSeek Genomic Profilers 3 the correlations of F_roh_ with the other estimators were increased from 0.89 to 0.95, 0.02 to 0.11, 0.61 to 0.69, 0.69 to 0.79, 0.29 to 0.60 and from 0.90 to 0.96 (for F, F_hat1_, F_hat2_, F_hat3_, F_grm_ and F_grm2_, respectively). Furthermore, there where cows with inbreeding coefficients close to 0 with the genotyped SNP, and high inbreeding coefficients with the imputed SNP. This was observed even with F_roh_ that was the most robust estimator. For instance, there was a group of cows genotyped with the GeneSeek Genomic Profiler 3 with inbreeding coefficients (based on genotyped SNP) ranging between ~0-0.15 while with imputation SNP the inbreeding coefficients were estimated between ~0.4 and 0.6 (Supplementary Figure 2C). Similar observation was made for cows genotyped with the GeneSeek Genomic Profiler 4 (Supplementary Figure 2D), where for few cows genotyped *f*_*SNP*_ ranged between 0 and 0.15 while the imputation *f*_*SNP*_ for those cows was >0.3. We could hypothesize that *f*_*SNP*_ estimated from ~15k SNP (as was the case of GeneSeek Genomic Profilers 3 and 4 in our study) might be biased. However, it must be also very unlikely that cows could have 40–60% of their genome in homozygous state, as was found with *f*_*SNP*_ estimated with imputation SNP. It is known that for a successful imputation three important components are (i) the distribution along the genome and the number of SNP in the LD/MD panels, (ii) the linkage disequilibrium between SNP in the MD and SNP in the HD (32–34) and (iii) the presence of genotypes from the parents and/or grandparents. For the GeneSeek Genomic Profilers 3 and 4, perhaps those could be hypothesized as limited parameters in our study.

## 5. Future perspectives

Passing through the second decade of applied genomic breeding programs, it is of interest that we still lack criteria to select a simple and optimal genomic inbreeding measure. In a recent work we showed that discrepancies among genomic inbreeding estimators exist (6) and some genomic inbreeding estimators can provide coefficients out of the range [-1, 1]; where negative coefficients reflect proportional gain of variability compared to a base unselected population of unrelated animals (5, 6, 35). Moreover, various parameters have to be considered when comparing genomic inbreeding estimators, such as SNP quality control, imputation methods, distinguishing between allozygosity – autozygosity, including SNP on the X-chromosome (28) to account for differences between males and females and better scale genomic inbreeding coefficients to pedigree inbreeding coefficients, to name some.

In the present study, we emphasized on the effect that imputation might have on the estimation of genomic inbreeding coefficients, relative to the density of SNP panels. Another important aspect of imputation relates to the relationship between animals genotyped with LD and MD SNP panels (to be imputed) and the animals that consist of the reference panel, and which were genotyped in HD. In a preliminary analysis, we have evidenced that indeed the correlation between genotyped – imputation *f*_*SNP*_ drastically degrades for the cows that have none of the parents and/or the maternal grand sire genotyped in HD and belonging in the reference set of the imputation pipeline (data not shown). This degrade in accuracy varies across the SNP panels and has been observed even with cows genotyped in HD, albeit to a much lower degree compared to cows genotyped in MD. This needs further investigation and quantification.

## 6. Conclusion

We investigated the effect of imputation, regarding the density of SNP panels used to genotype cows, in a routine dairy cattle genomic breeding program on SNP inbreeding coefficients. Correlations between genotyped vs. imputation SNP inbreeding coefficients were high and consistent for the HD SNP panels. Accuracies were degraded for the four MD density SNP panels. This drop in accuracy was linked to the number of SNP of the SNP panel included in the imputation SNP set and the average distance of SNP on the genome. Assuming that high correlation with pedigree inbreeding coefficients reflects more realistic values, genomic inbreeding coefficients estimated from imputation SNP were biased for the cows genotyped with MD SNP panels, because they were less correlated to pedigree inbreeding coefficients compared to inbreeding coefficients estimated only from genotyped SNP.

We wish to state that our analysis is not critical on the quality of commercial SNP panels *per se*, but rather it highlights the effect that the imputation pipeline and the overall genotyping management might have on the genomic inbreeding coefficients. Our results indicate that SNP panels that contain more informative SNP for the population under study can have more genotyped SNP that remain in the imputation data and thereby provide with more robust results on the genomic inbreeding coefficients of the cows. Cows that were genotyped with MD SNP panels that had few SNP included in the final imputed SNP data were more likely to have biased genomic inbreeding estimates for some groups of cows. In such a concept, F_roh_ can be considered as a more robust estimator, reflecting identity-by-descent, compared to estimators summing homozygosity over individual SNP, measuring identity-by-state.

## Data availability statement

The data analyzed in this study is subject to the following licenses/restrictions: Data supporting this paper were obtained from ANAFIBJ. The genotype data are available only upon agreement with ANAFIBJ. Requests to access these datasets should be directed to J-TK, jtkaam@anafi.it.

## Ethics statement

Ethical review and approval was not required for the animal study because no animals were used in this study, and ethical approval for the use of animals was thus deemed unnecessary.

## Author contributions

CD, J-TK, CC-G, and MA conceived the idea and formulated the objectives of this study. J-TK helped in data preparation. CD conducted the analysis and wrote the first draft of the paper. ASu and ASa supervised the project. ASu, CC-G, ASa, MM, RF, and MC critically reviewed the text. All authors read and approved the final manuscript.

## Supplementary material

Supplementary Figure 1 SNP density per chromosome for the **(A)** imputed, **(B)** Illumina Infinium BovineHD BeadChip, **(C)** GeneSeek Genomic Profiler HD-150K, **(D)** GeneSeek Genomic Profiler 3, **(E)** GeneSeek Genomic Profiler 4, **(F)** GeneSeek MD, and **(G)** Labogena MD.

Supplementary Figure 2 Comparison between genotyped and imputed SNP inbreeding coefficients for **(A)** Illumina Infinium BovineHD BeadChip, **(B)** GeneSeek GenomicProfiler HD-150K, **(C)** GeneSeek Genomic Profiler 3, **(D)** GeneSeek Genomic Profiler 4, **(E)** GeneSeek MD, and **(F)** Labogena MD.

Supplementary Figure 3 Pairwise Pearson correlations (above diagonal) between each pair of the pedigree and the seven genomic inbreeding estimators analyzed for **(A)** Illumina Infinium BovineHD BeadChip, **(B)** GeneSeek Genomic Profiler HD-150K, **(C)** GeneSeek Genomic Profiler 3, **(D)** GeneSeek Genomic Profiler 4, **(E)** GeneSeek MD, and **(F)** Labogena MD. In gray color the overall correlation, in red correlations estimated from the genotyped SNP in each panel and in green correlations estimated from the imputation SNP.
