# Supplementary material for: Genomic inbreeding coefficients using imputed genotypes: assessing differences among SNP panels in Holstein-Friesian dairy cows
Source: Front Vet Sci. 2023 Apr 28;10:1142476. doi: 10.3389/fvets.2023.1142476 (PMC10180025; doi:10.3389/fvets.2023.1142476)
Supplement: Supplementary Figure 1 — SNP density per chromosome for the (A) imputed, (B) Illumina Infinium BovineHD BeadChip, (C) GeneSeek Genomic Profiler HD-150K, (D) GeneSeek Genomic Profiler 3, (E) GeneSeek Genomic Profiler 4, (F) GeneSeek MD, and (G) Labogena MD. [file Data_Sheet_1.PDF]

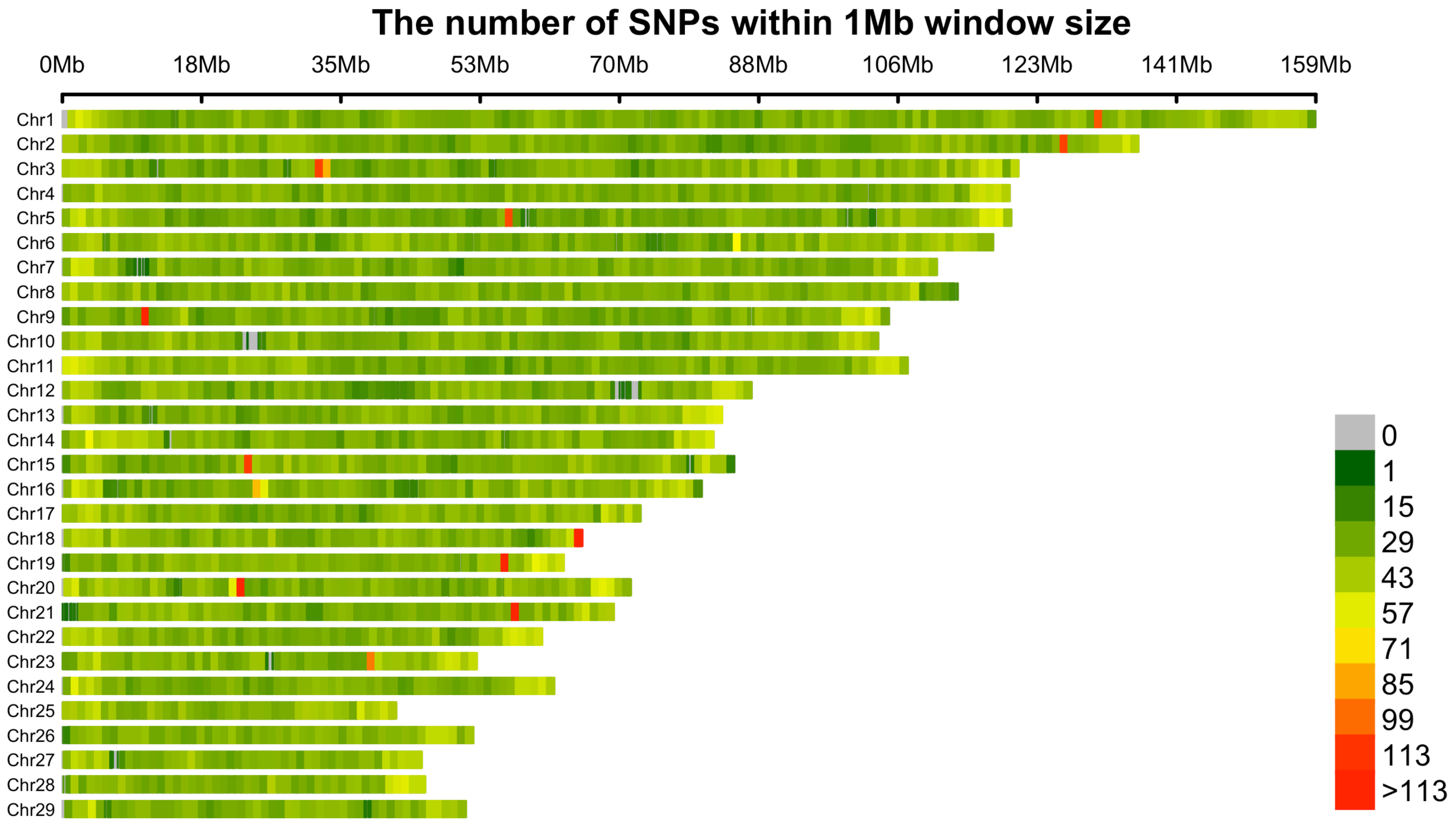

a) Imputation SNP

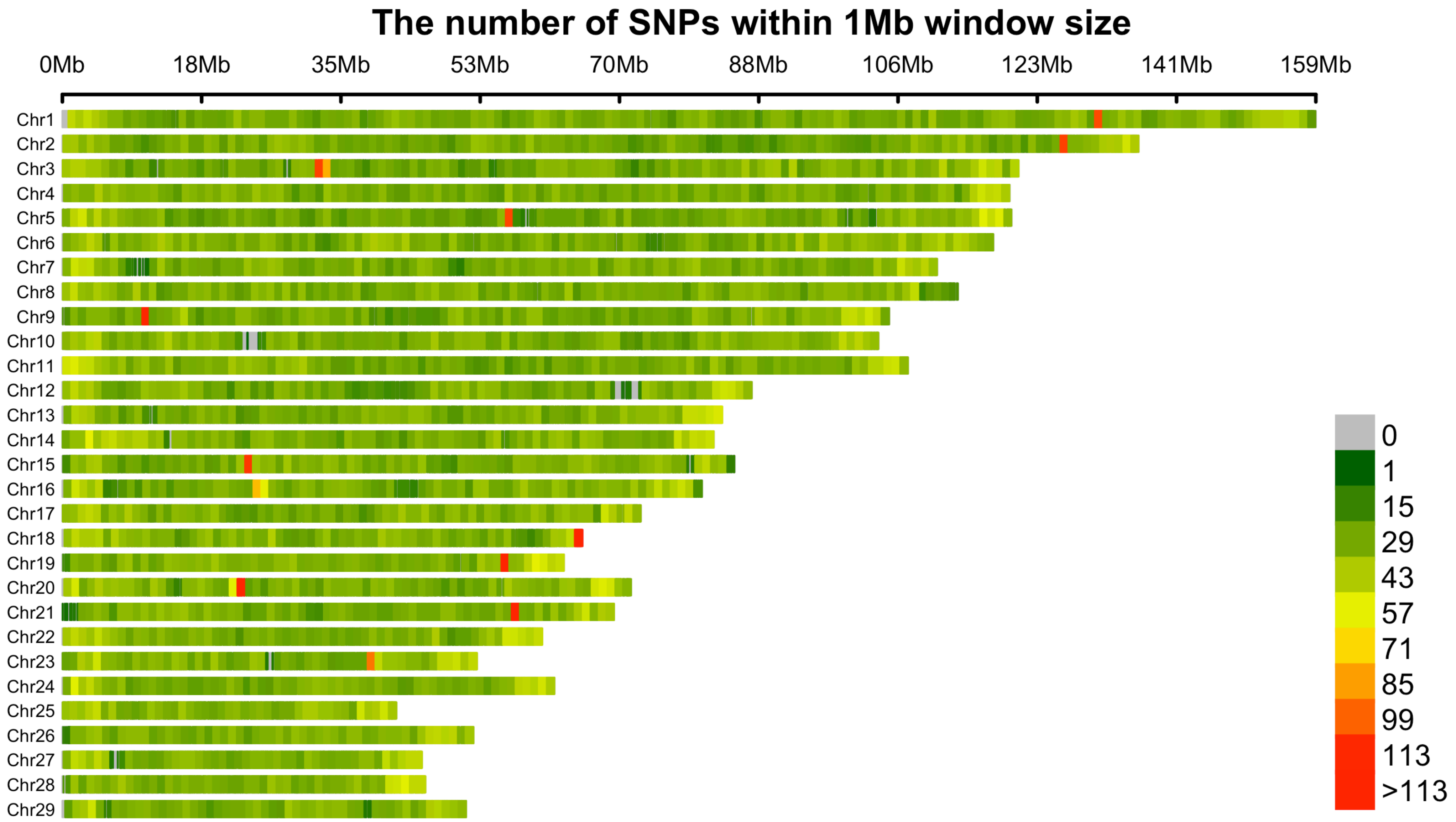

b) Illumina Infinium BovineHD BeadChip

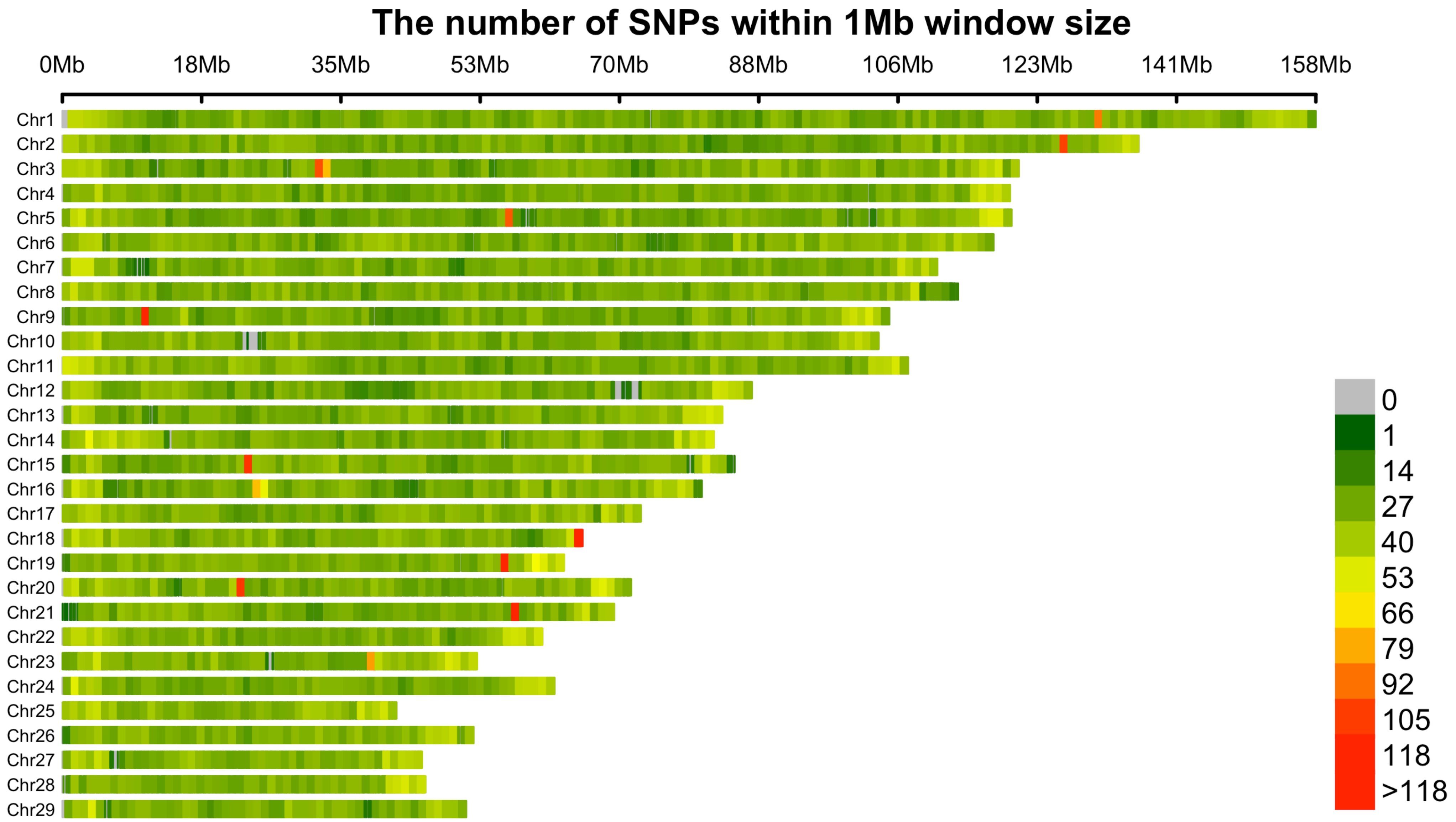

c) GeneSeek Genomic Profiler HD-150K

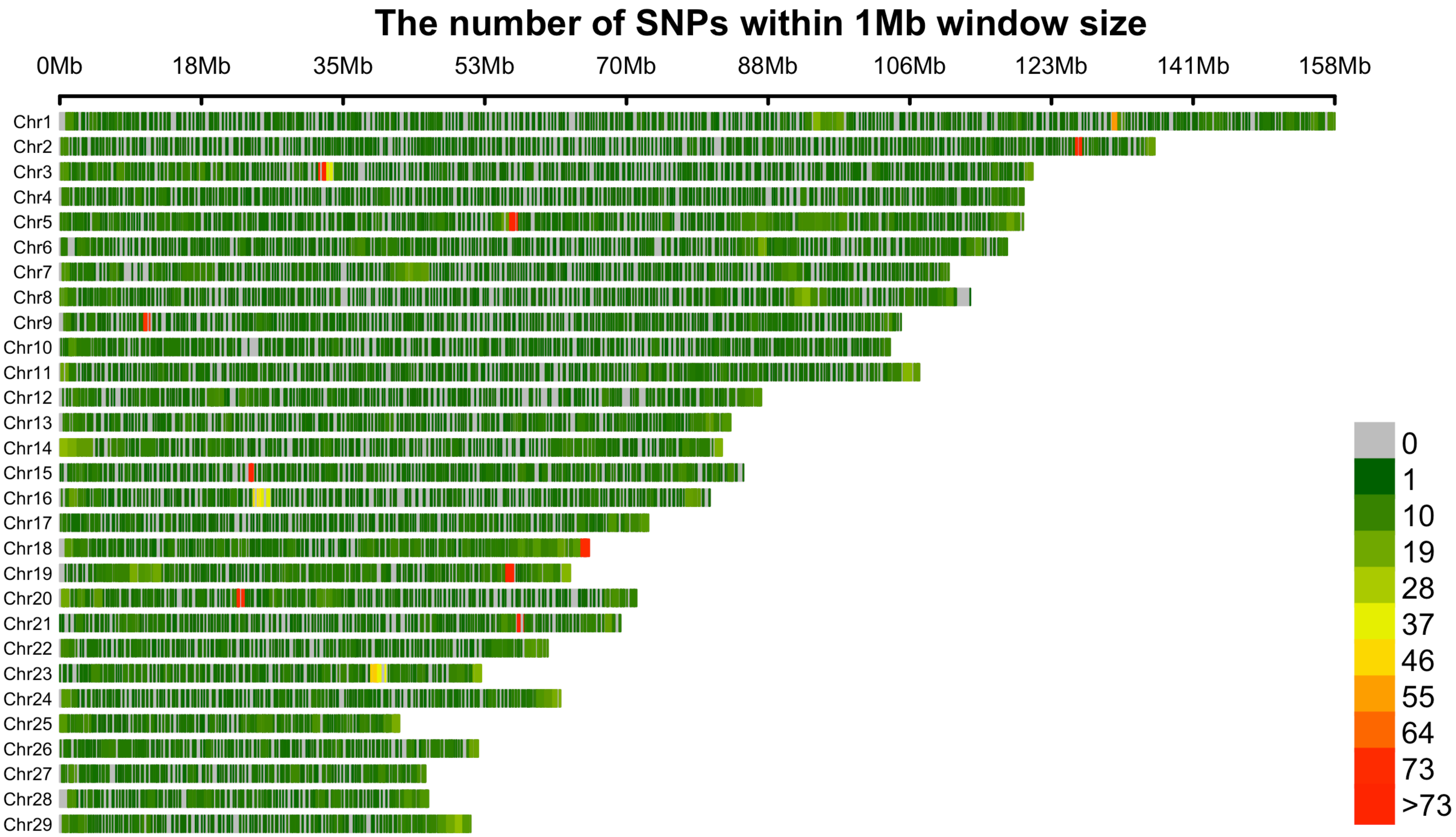

d) GeneSeek Genomic Profiler 3

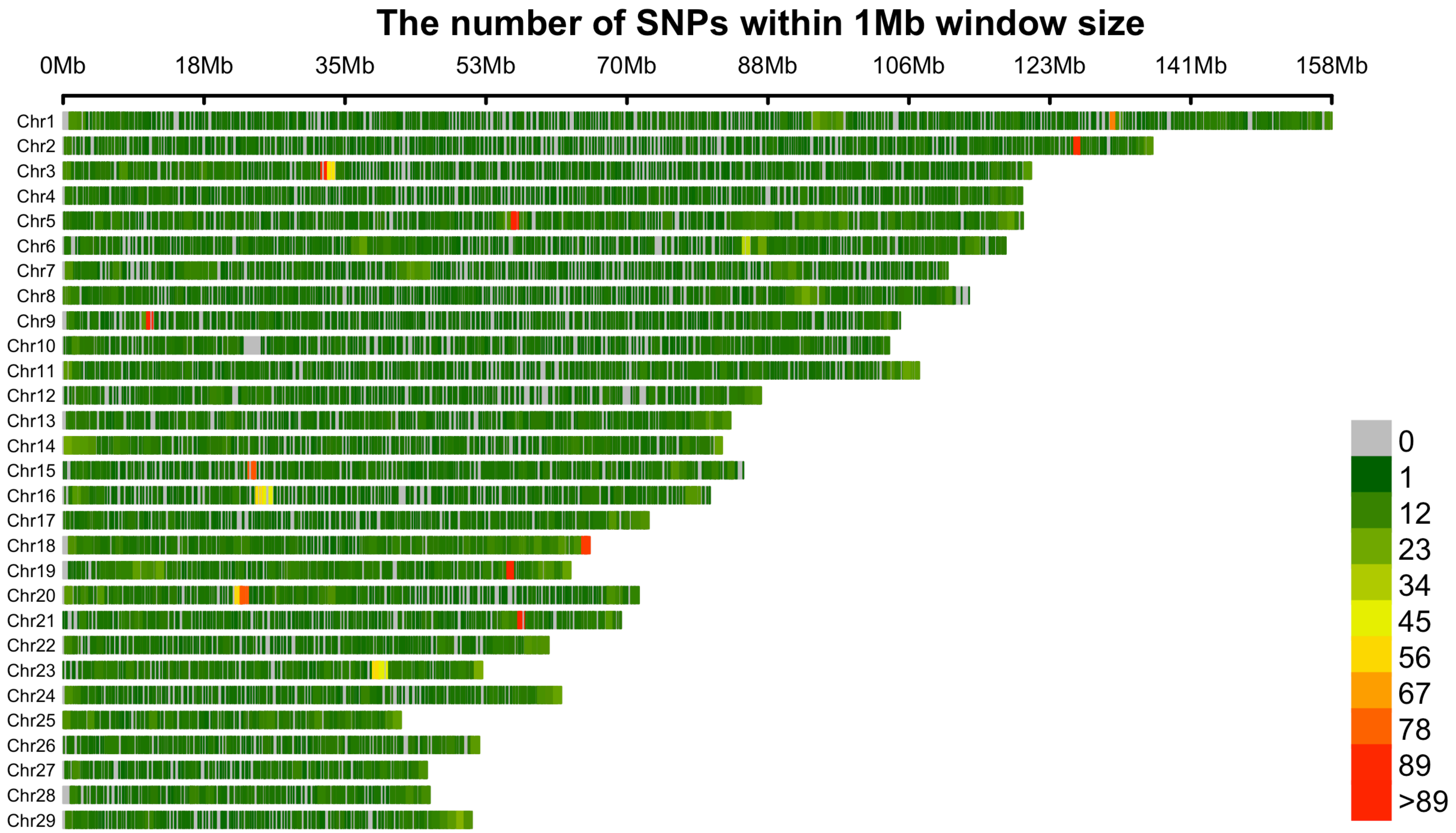

e) GeneSeek Genomic Profiler 4

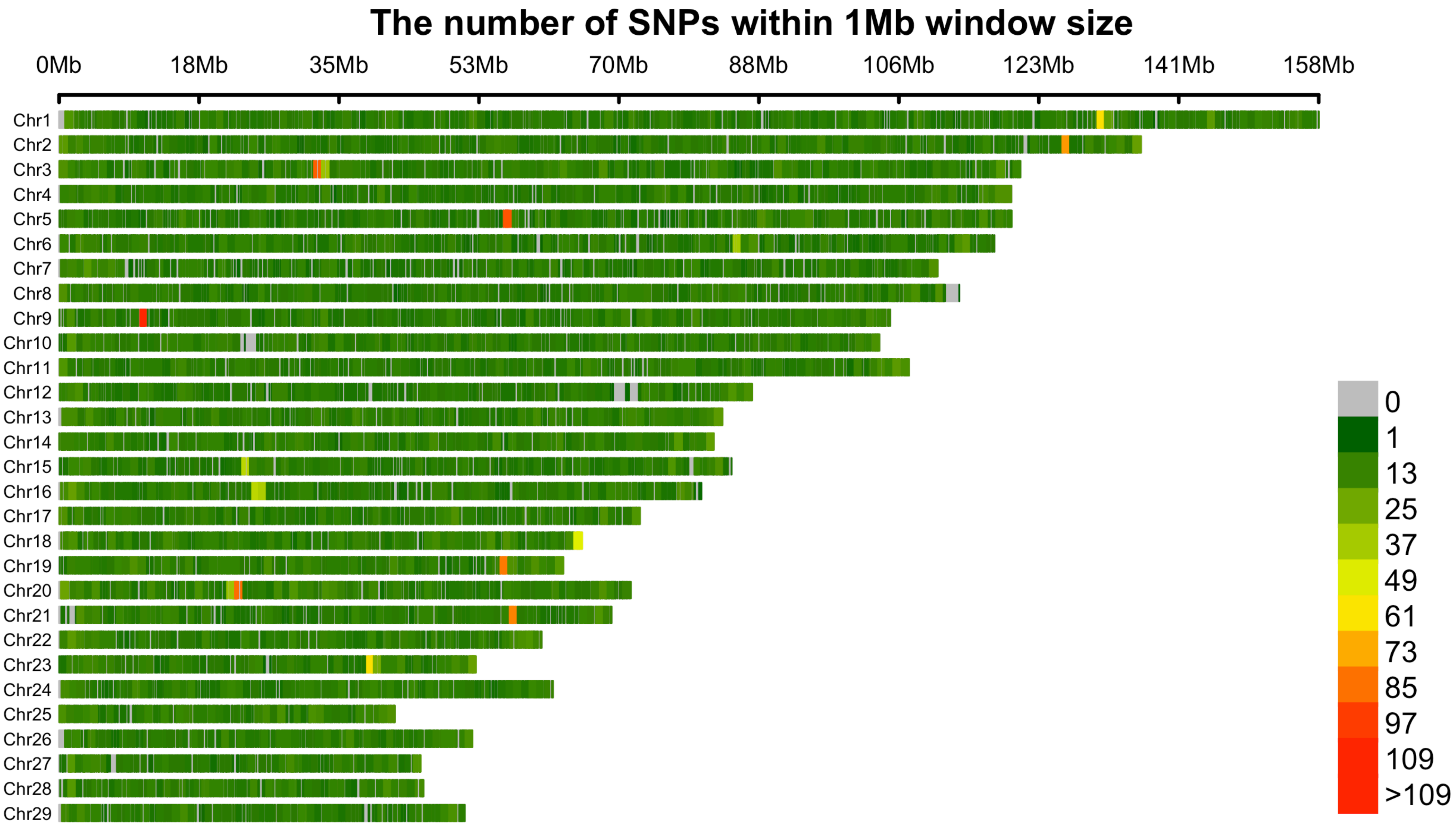

f) GeneSeek MD

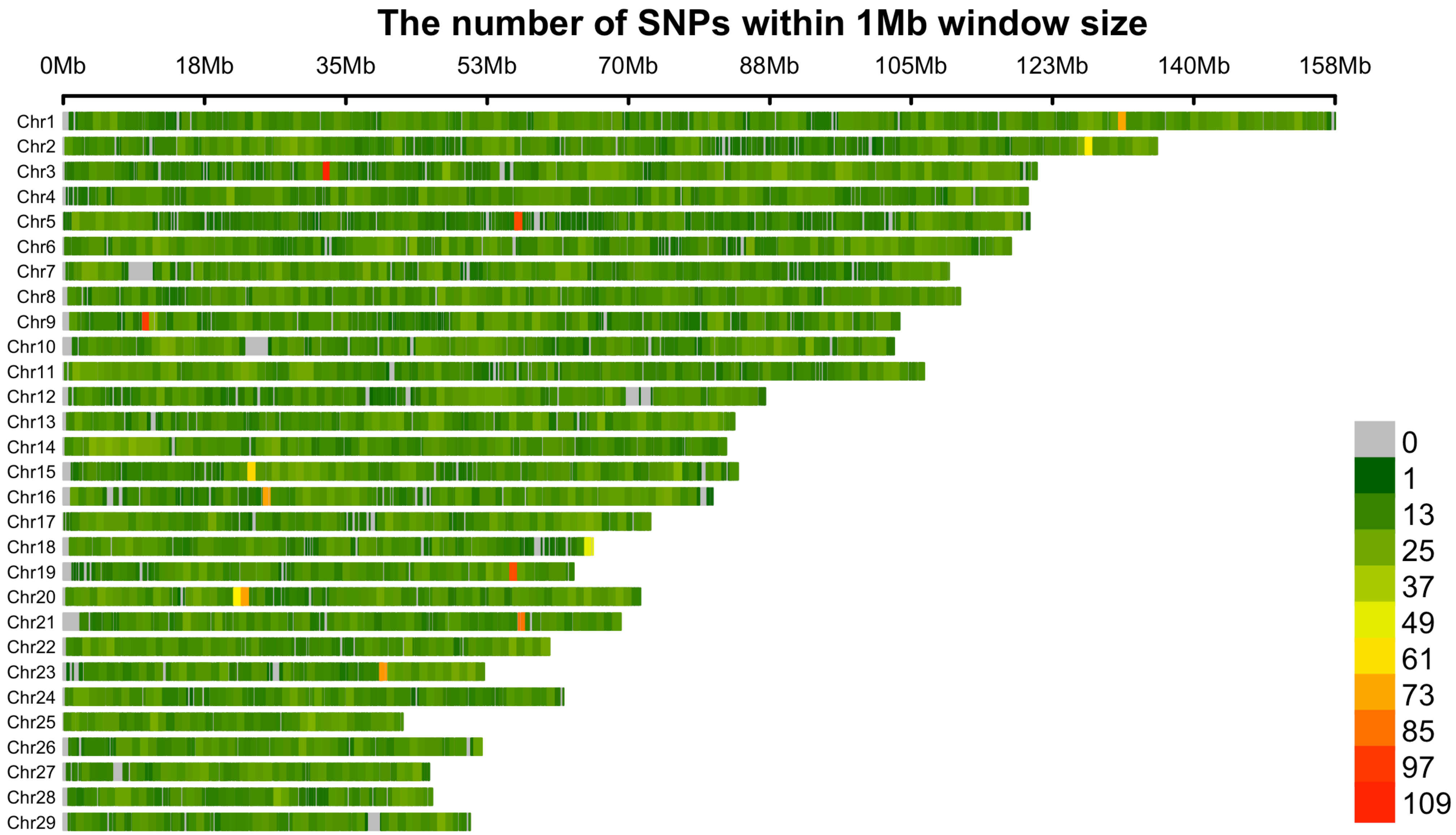

g) Labogena MD
