# Supplementary figures and images for: Genomic inbreeding coefficients using imputed genotypes: assessing differences among SNP panels in Holstein-Friesian dairy cows
Source: Front Vet Sci. 2023 Apr 28;10:1142476. doi: 10.3389/fvets.2023.1142476 (PMC10180025; doi:10.3389/fvets.2023.1142476)

Illumina Infinium BovineHD BeadChip

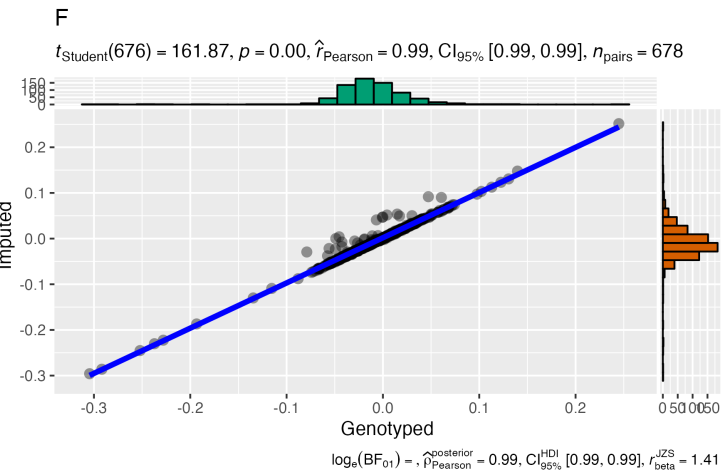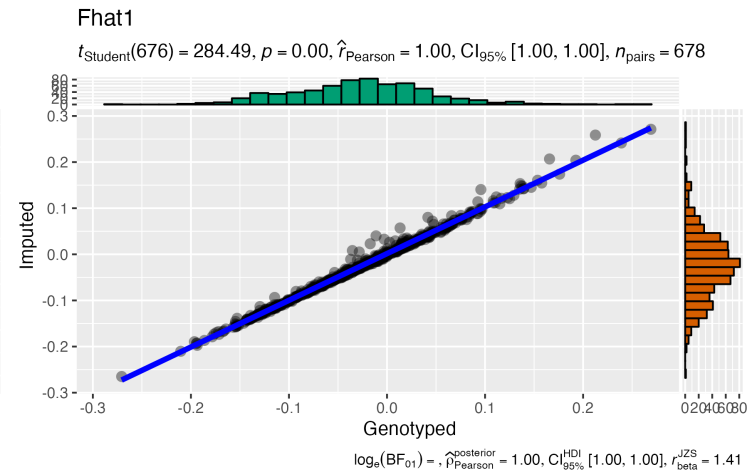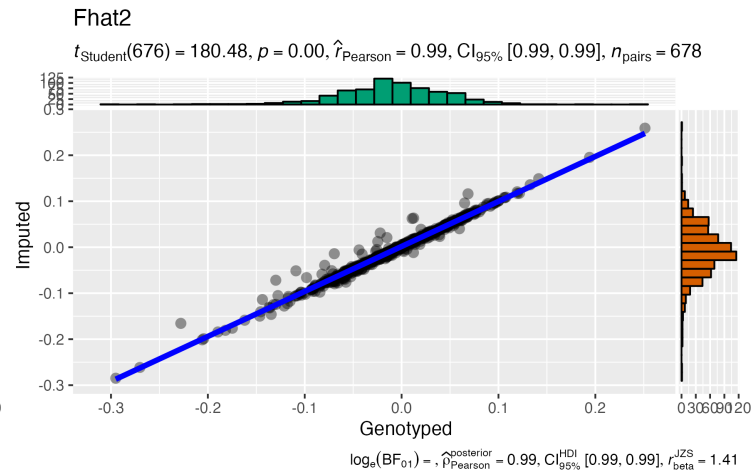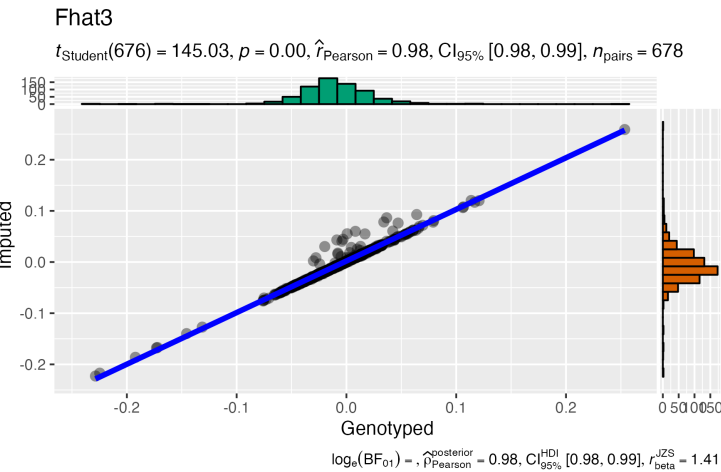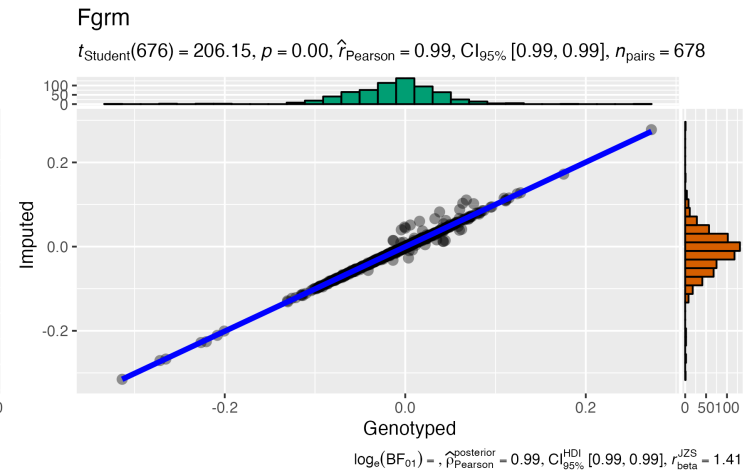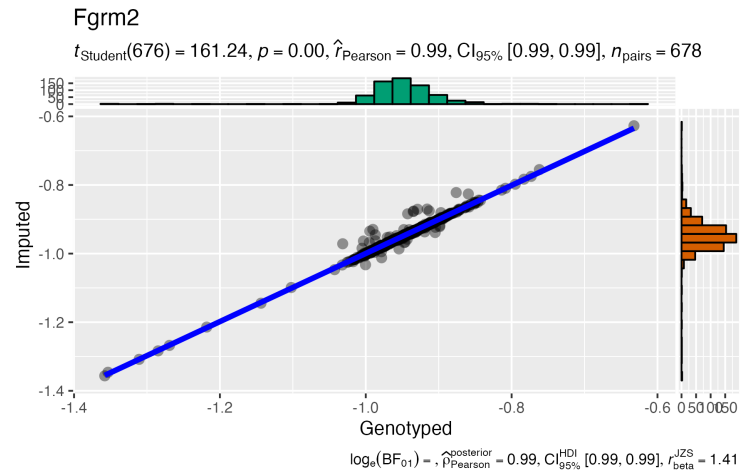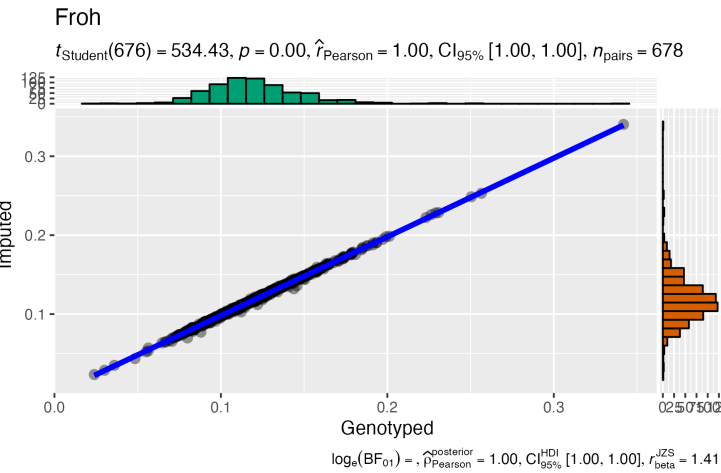

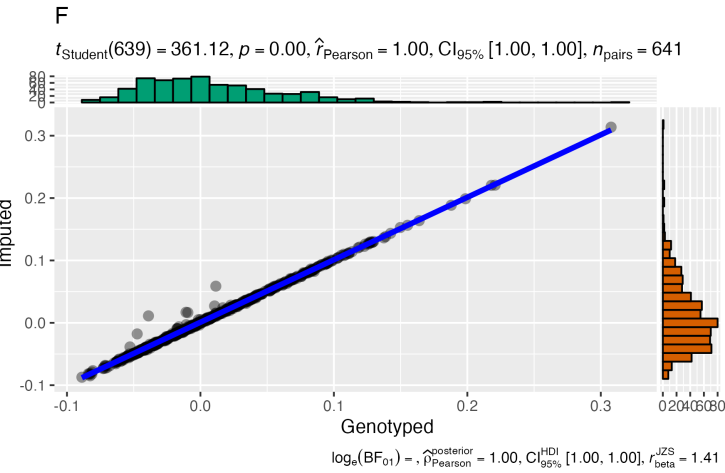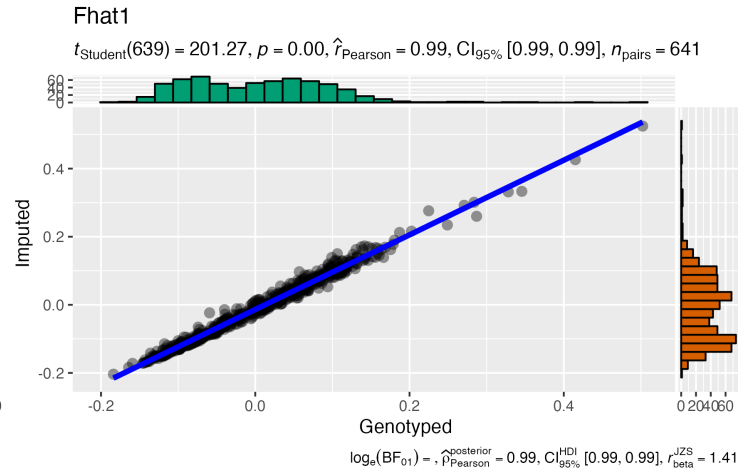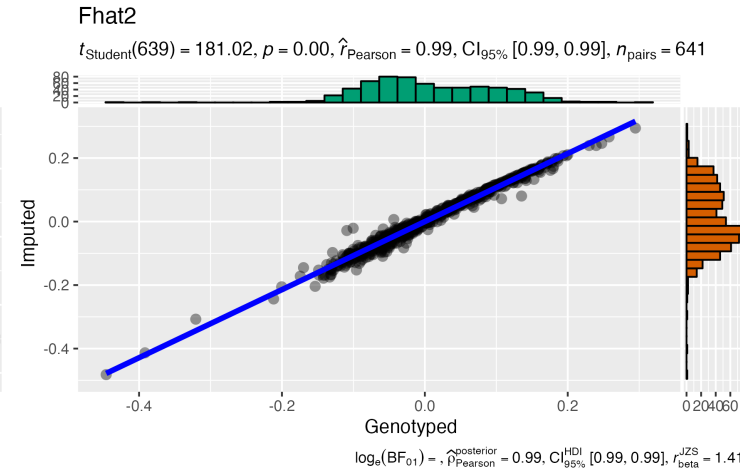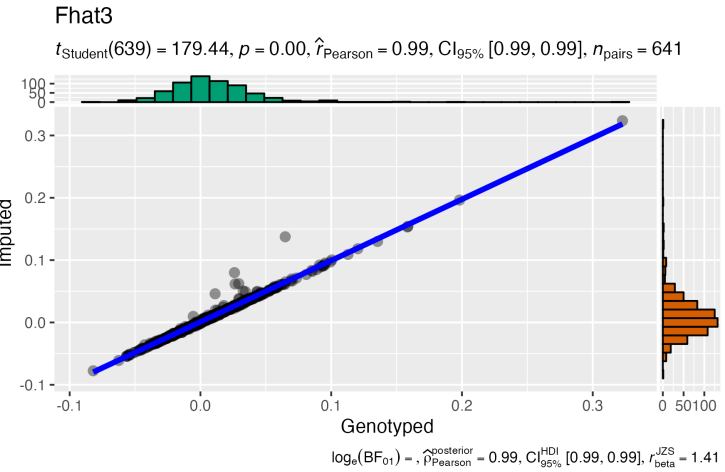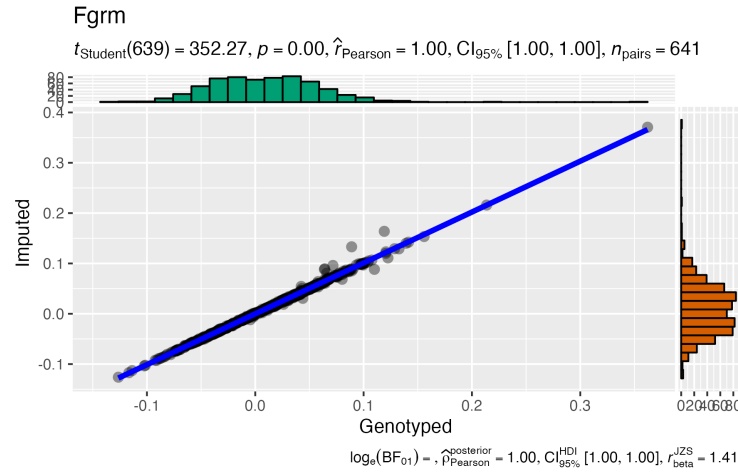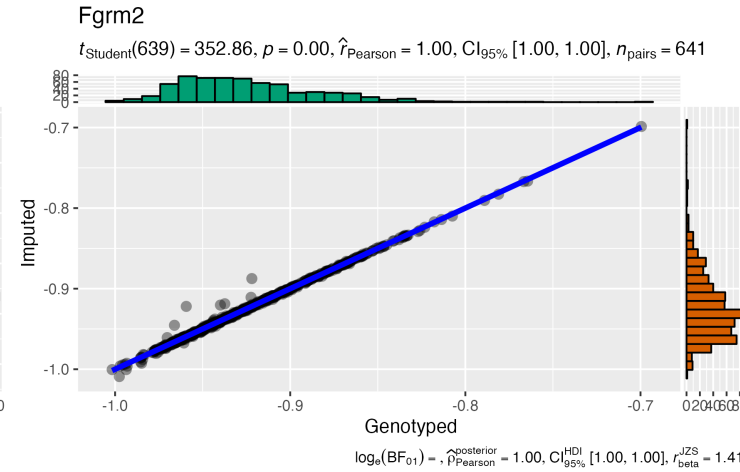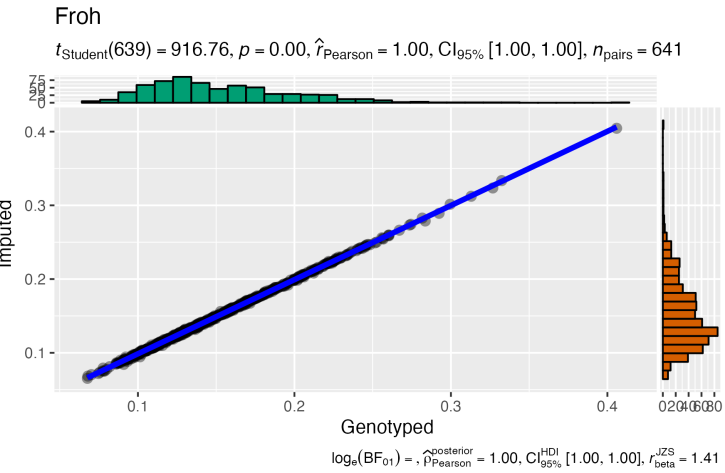

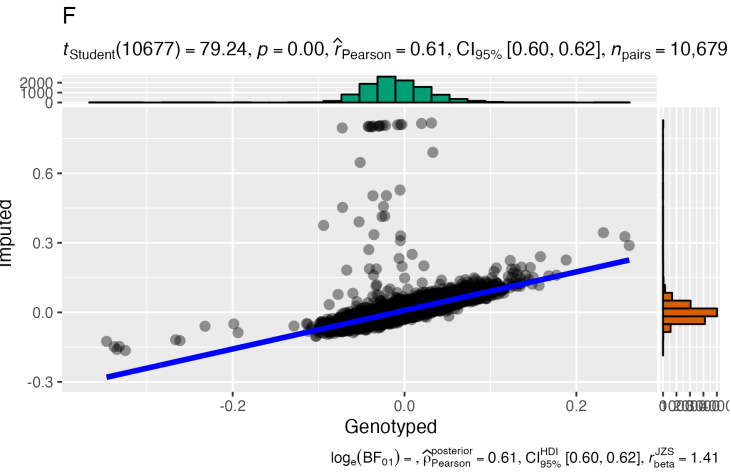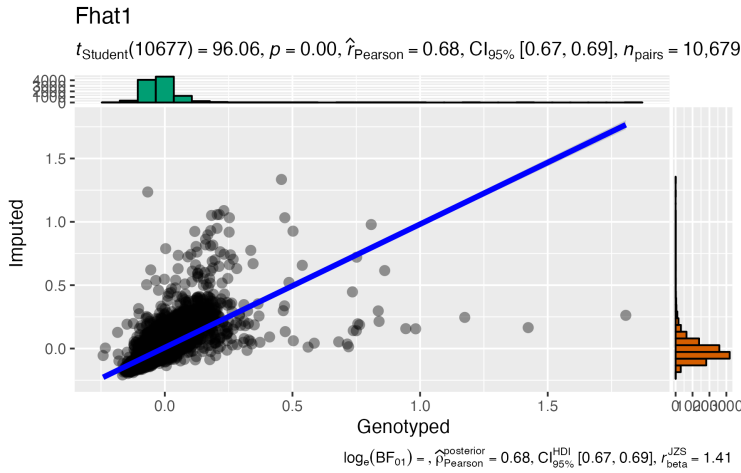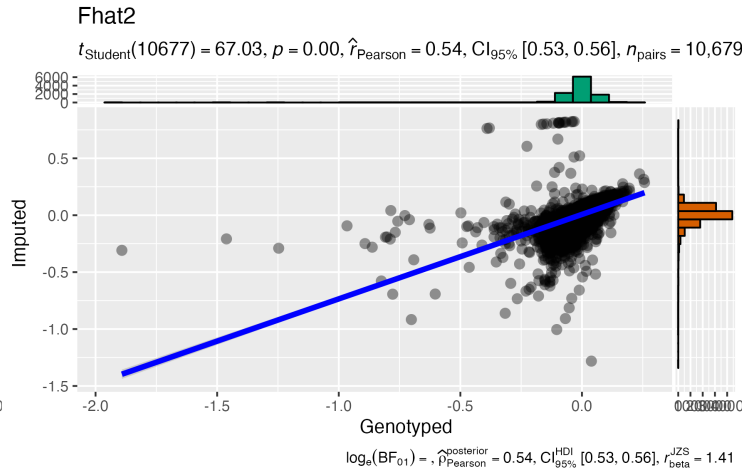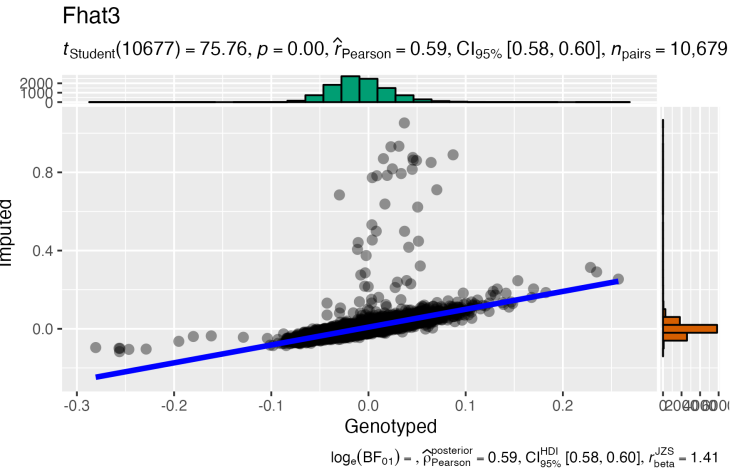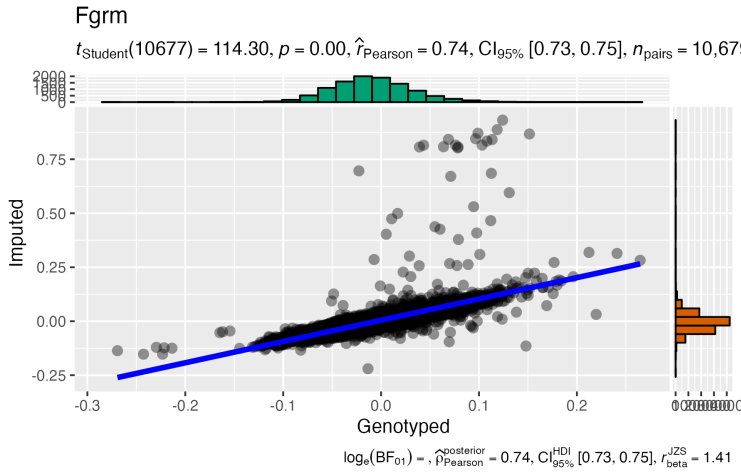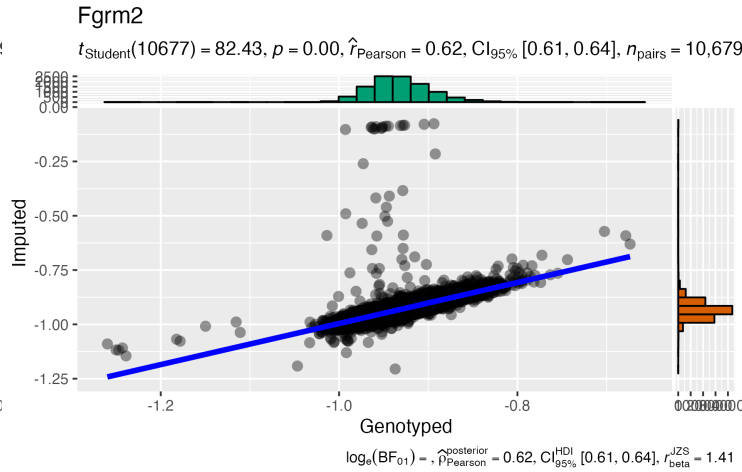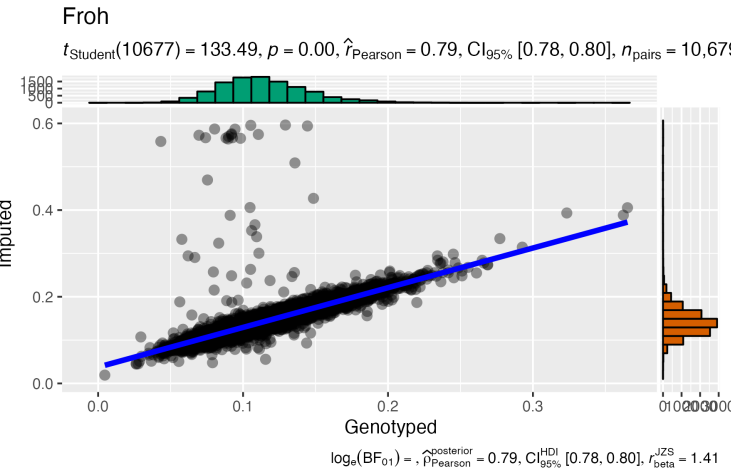

GeneSeek Genomic Profiler 4

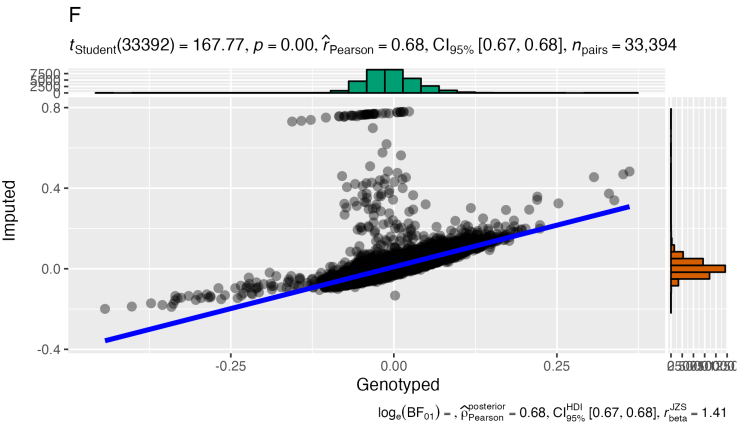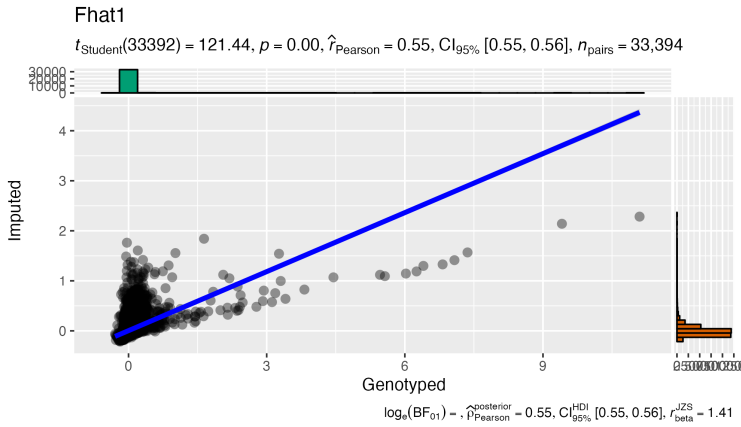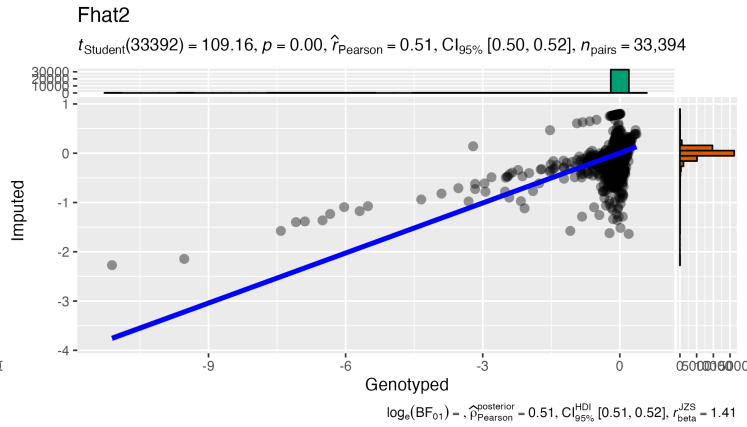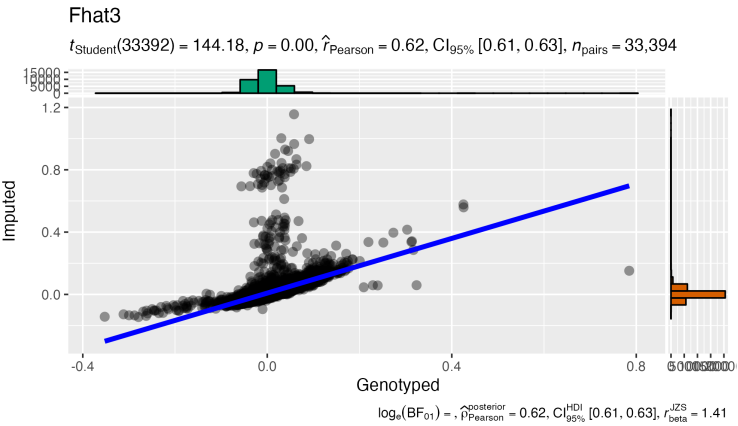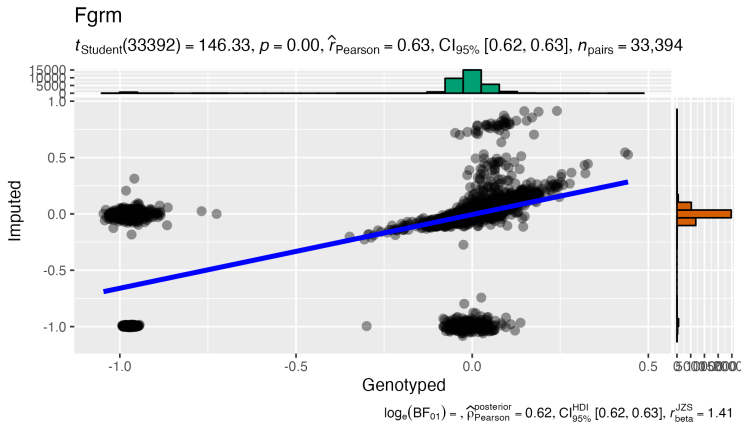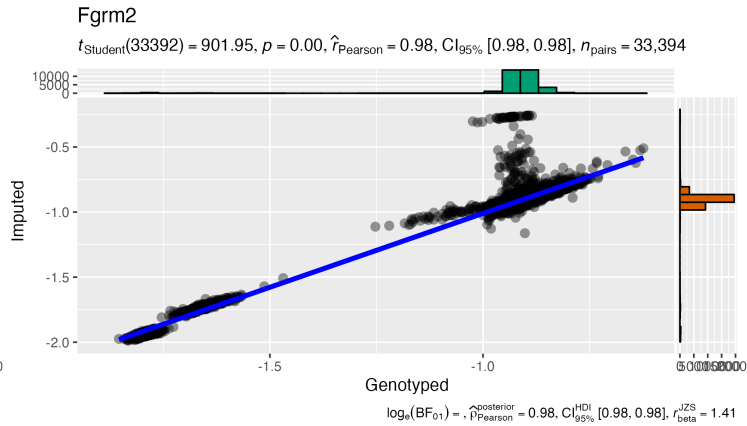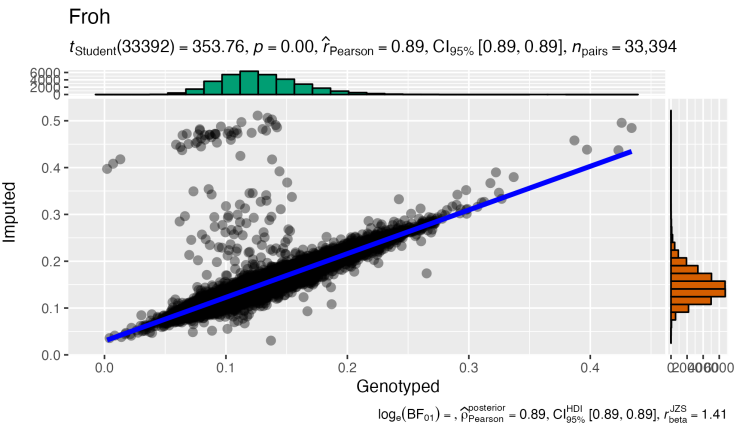

GeneSeek MD

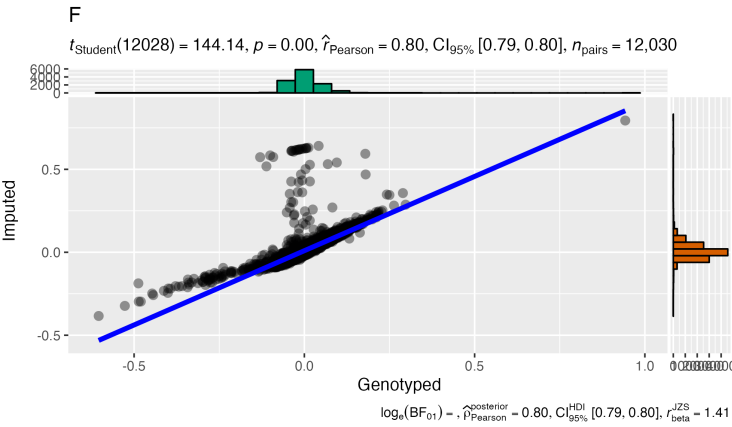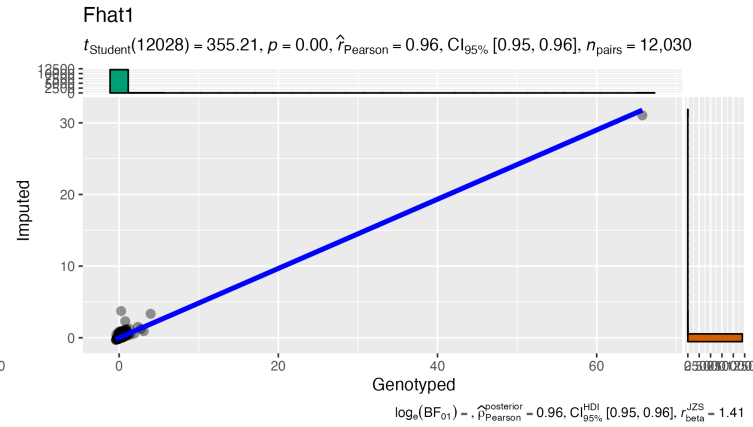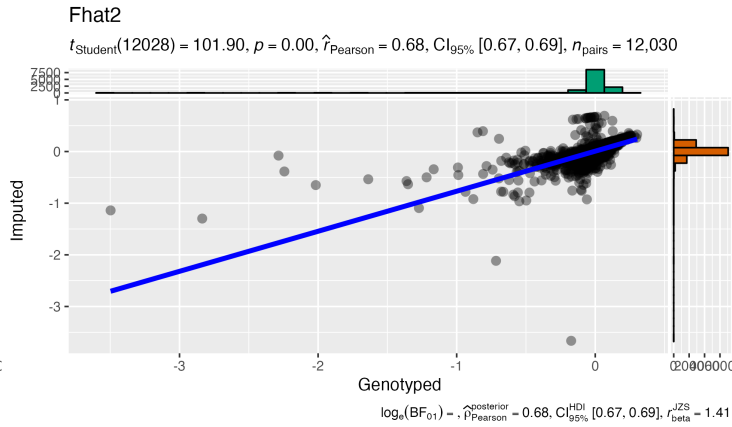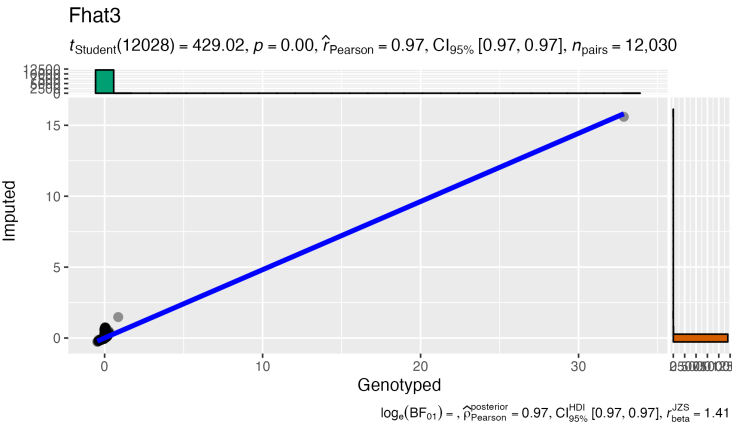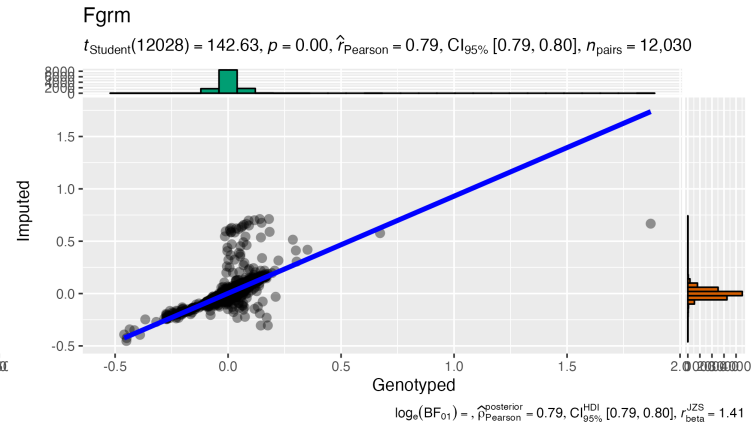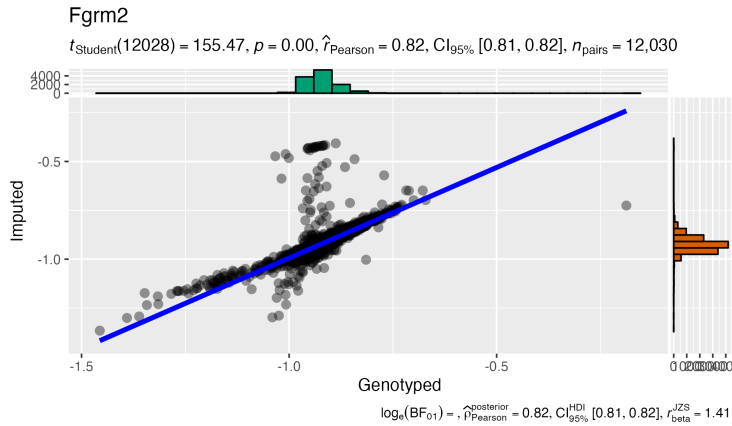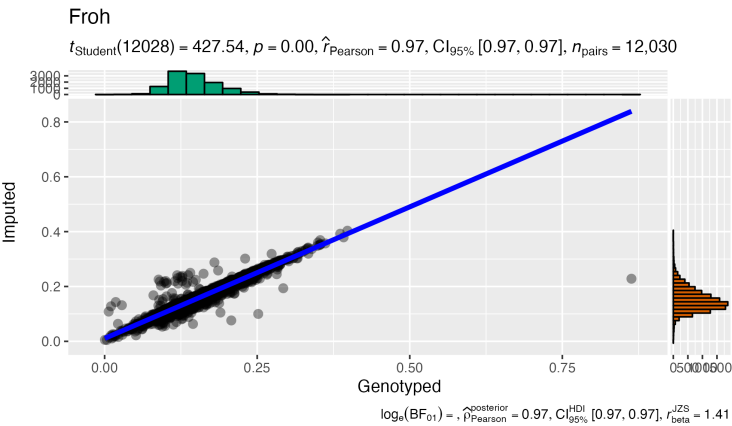

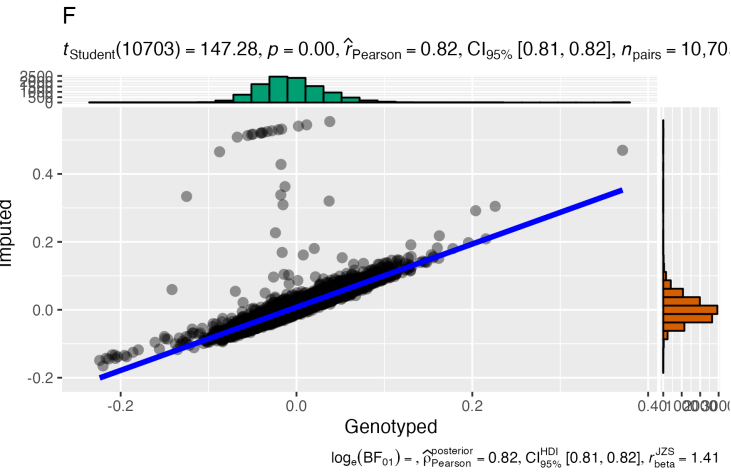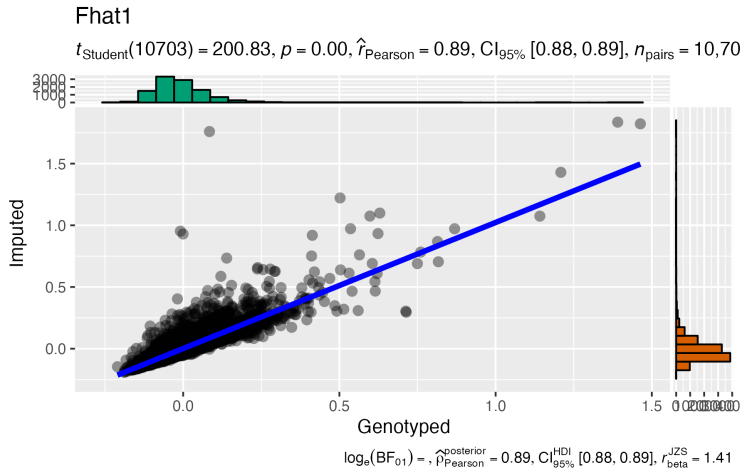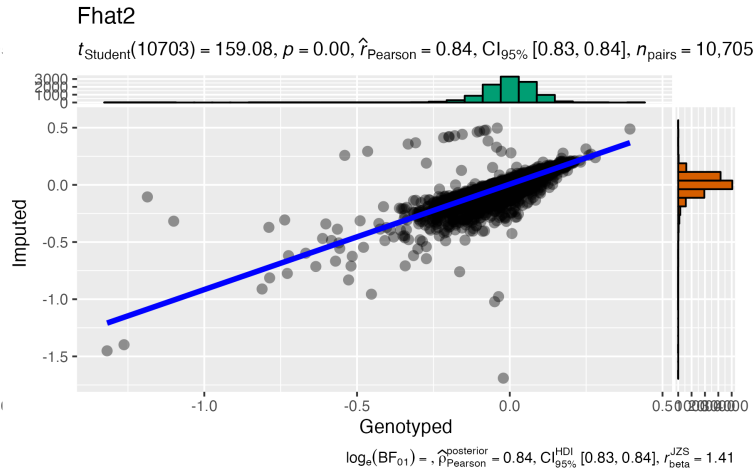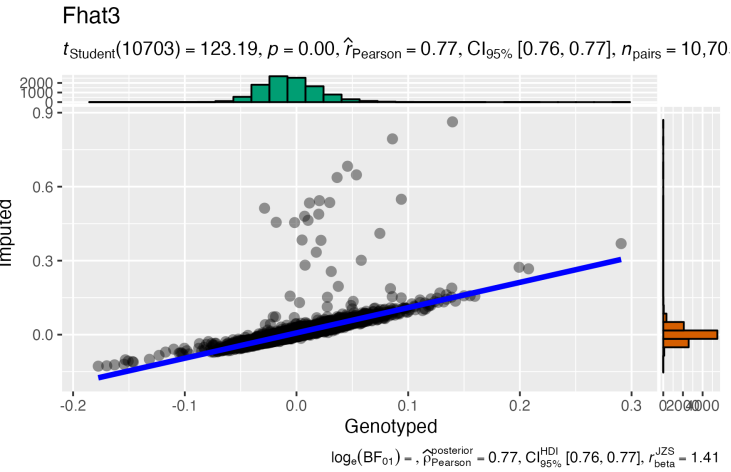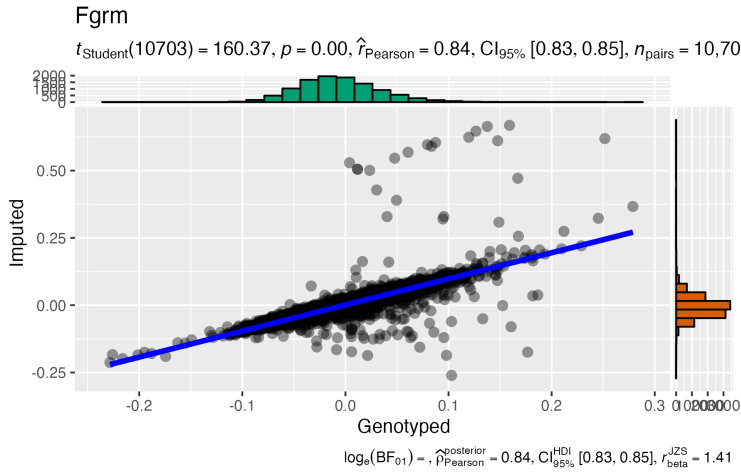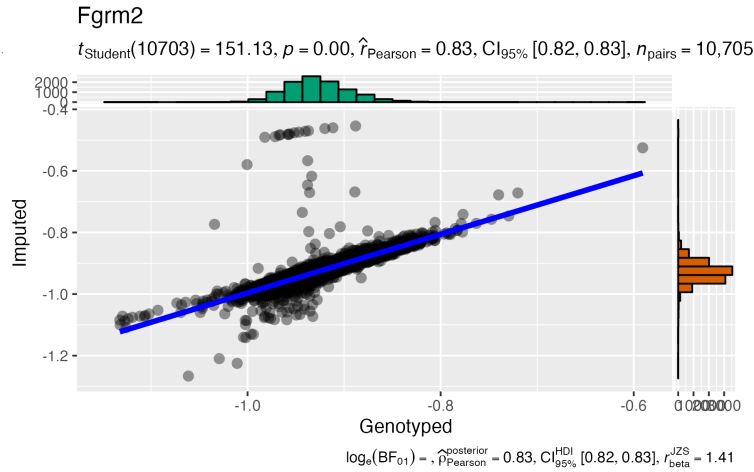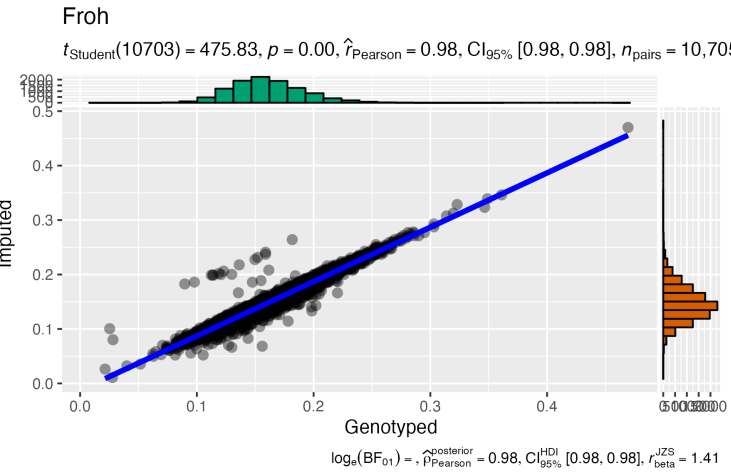

Supplement: Supplementary Figure 2 — Comparison between genotyped and imputed SNP inbreeding coefficients for (A) Illumina Infinium BovineHD BeadChip, (B) GeneSeek GenomicProfiler HD-150K, (C) GeneSeek Genomic Profiler 3, (D) GeneSeek Genomic Profiler 4, (E) GeneSeek MD, and (F) Labogena MD. [file Data_Sheet_2.PDF]
