# Supplementary material for: Genomic inbreeding coefficients using imputed genotypes: assessing differences among SNP panels in Holstein-Friesian dairy cows
Source: Front Vet Sci. 2023 Apr 28;10:1142476. doi: 10.3389/fvets.2023.1142476 (PMC10180025; doi:10.3389/fvets.2023.1142476)
Supplement: Supplementary Figure 3 — Pairwise Pearson correlations (above diagonal) between each pair of the pedigree and the seven genomic inbreeding estimators analyzed for (A) Illumina Infinium BovineHD BeadChip, (B) GeneSeek Genomic Profiler HD-150K, (C) GeneSeek Genomic Profiler 3, (D) GeneSeek Genomic Profiler 4, (E) GeneSeek MD, and (F) Labogena MD. In gray color the overall correlation, in red correlations estimated from the genotyped SNP in each panel and in green correlations estimated from the imputation SNP. [file Data_Sheet_3.PDF]

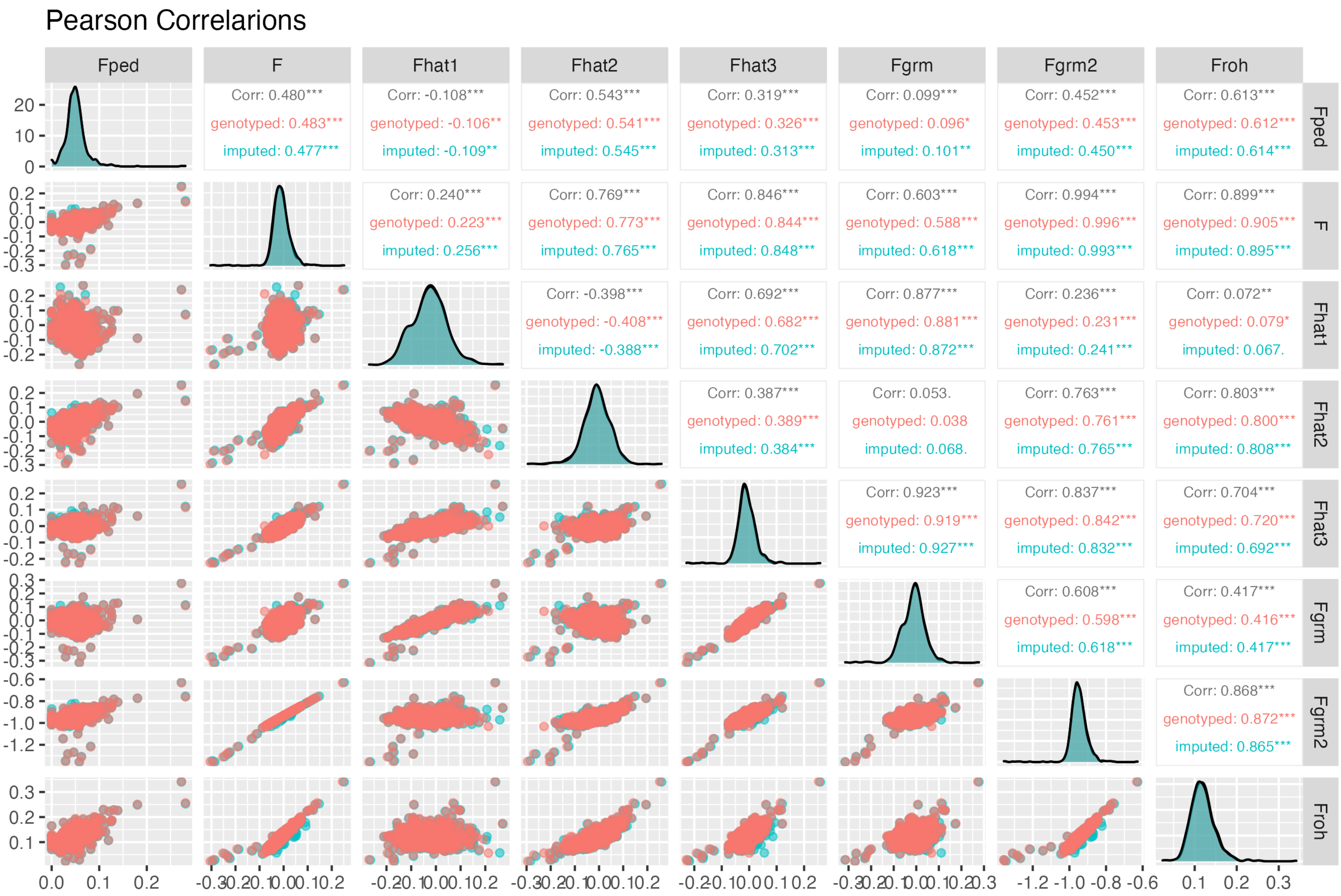

a) Illumina Infinium BovineHD BeadChip

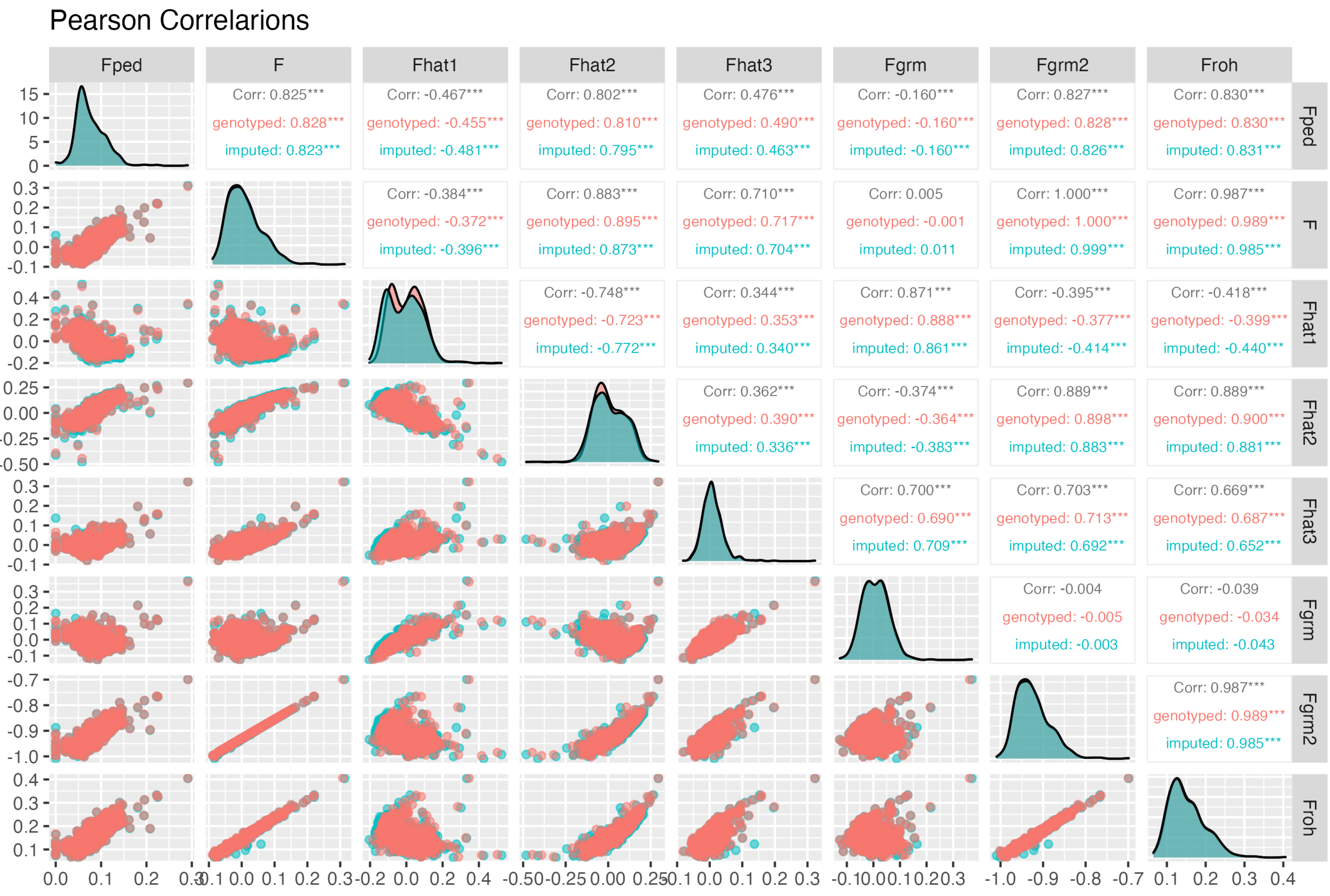

b) GeneSeek Genomic Profiler HD-150K

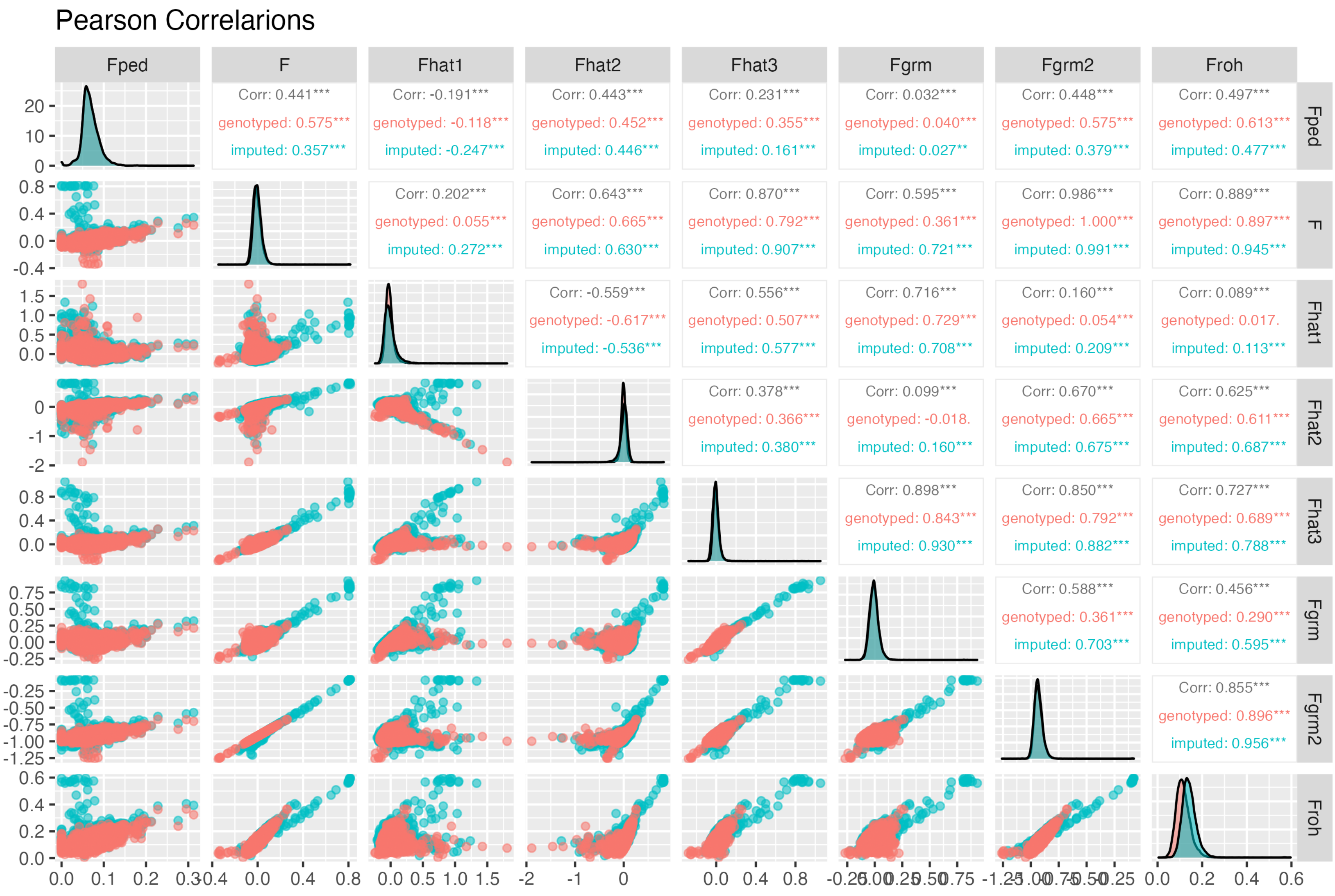

c) GeneSeek Genomic Profiler 3

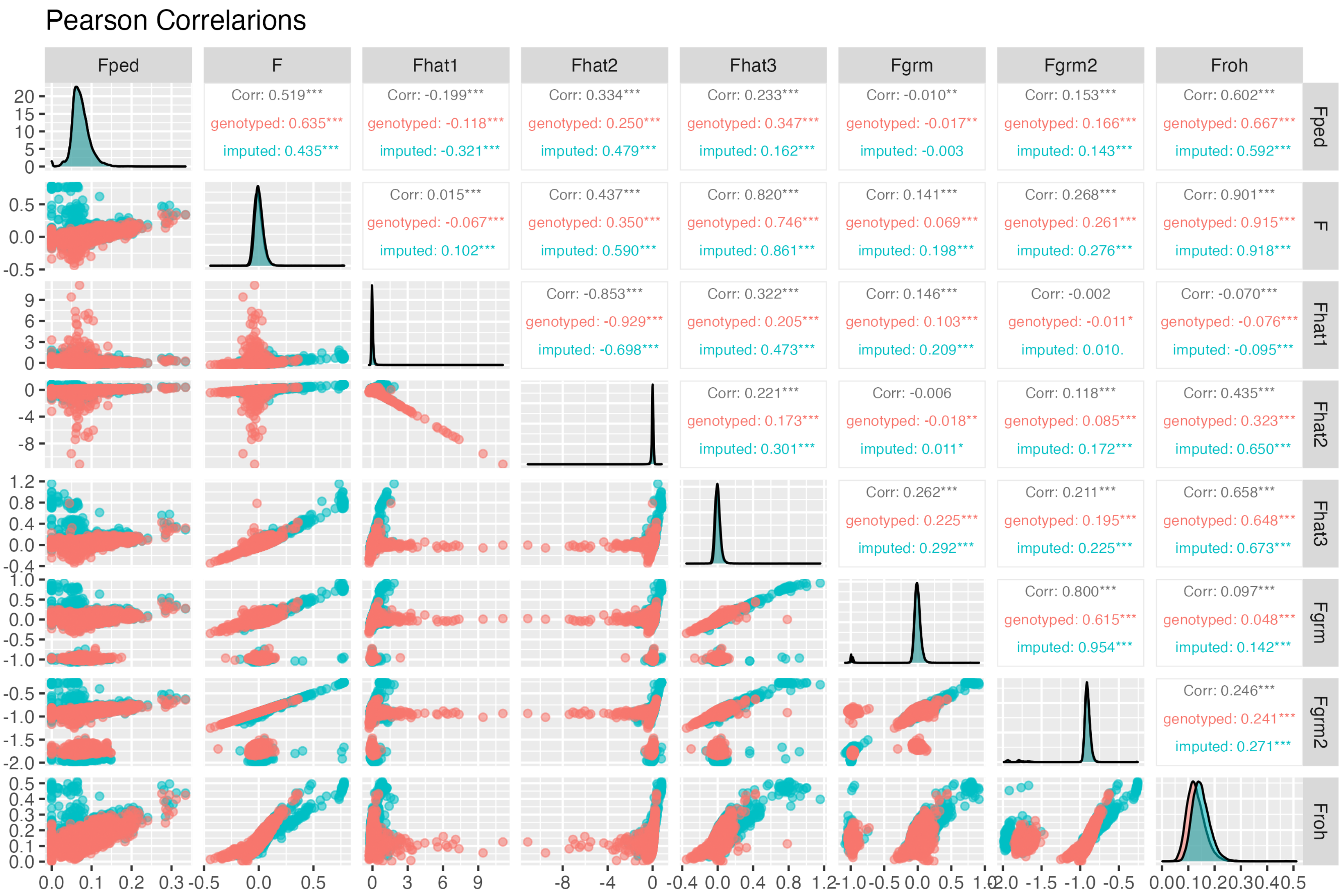

d) GeneSeek Genomic Profiler 4

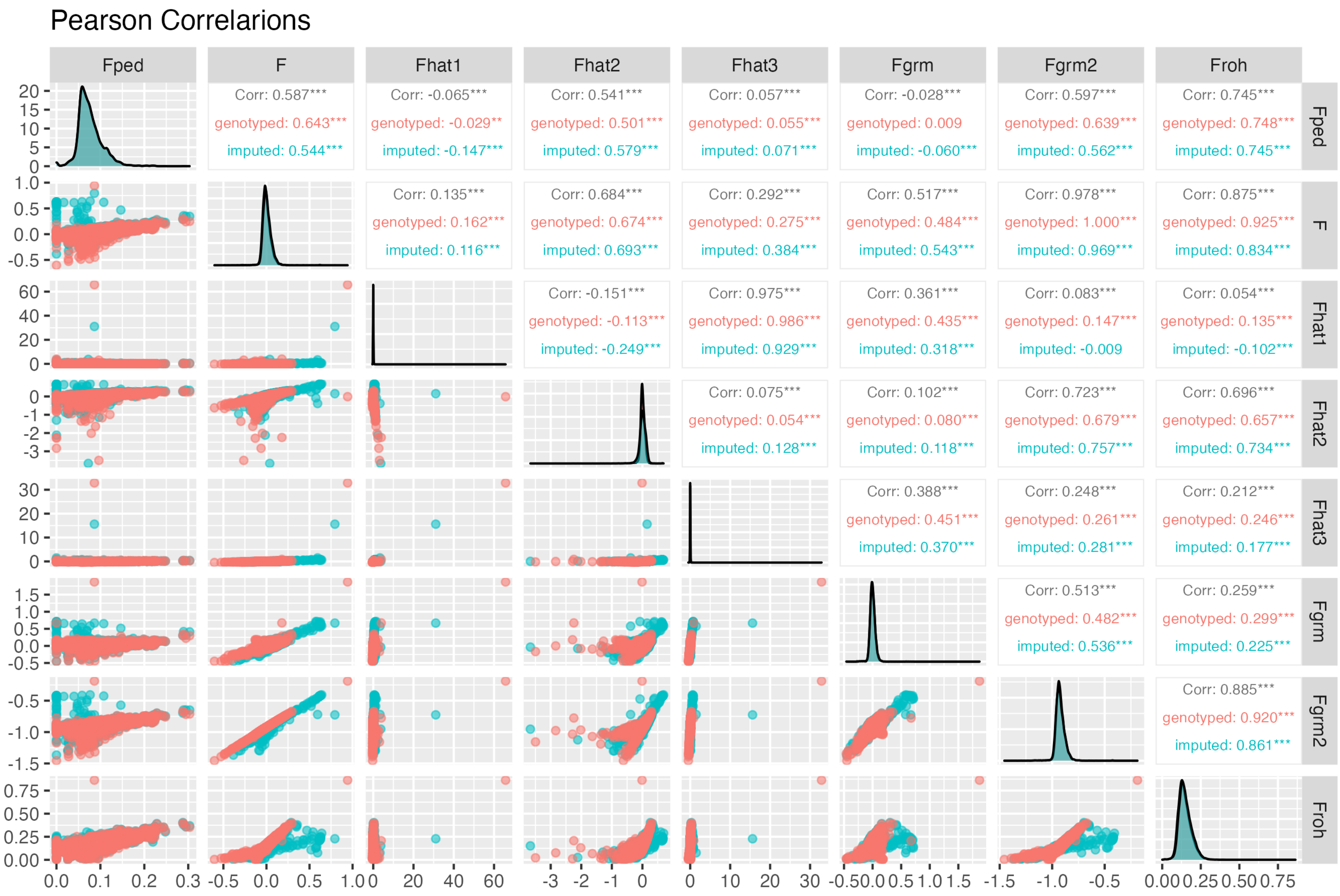

e) GeneSeek MD

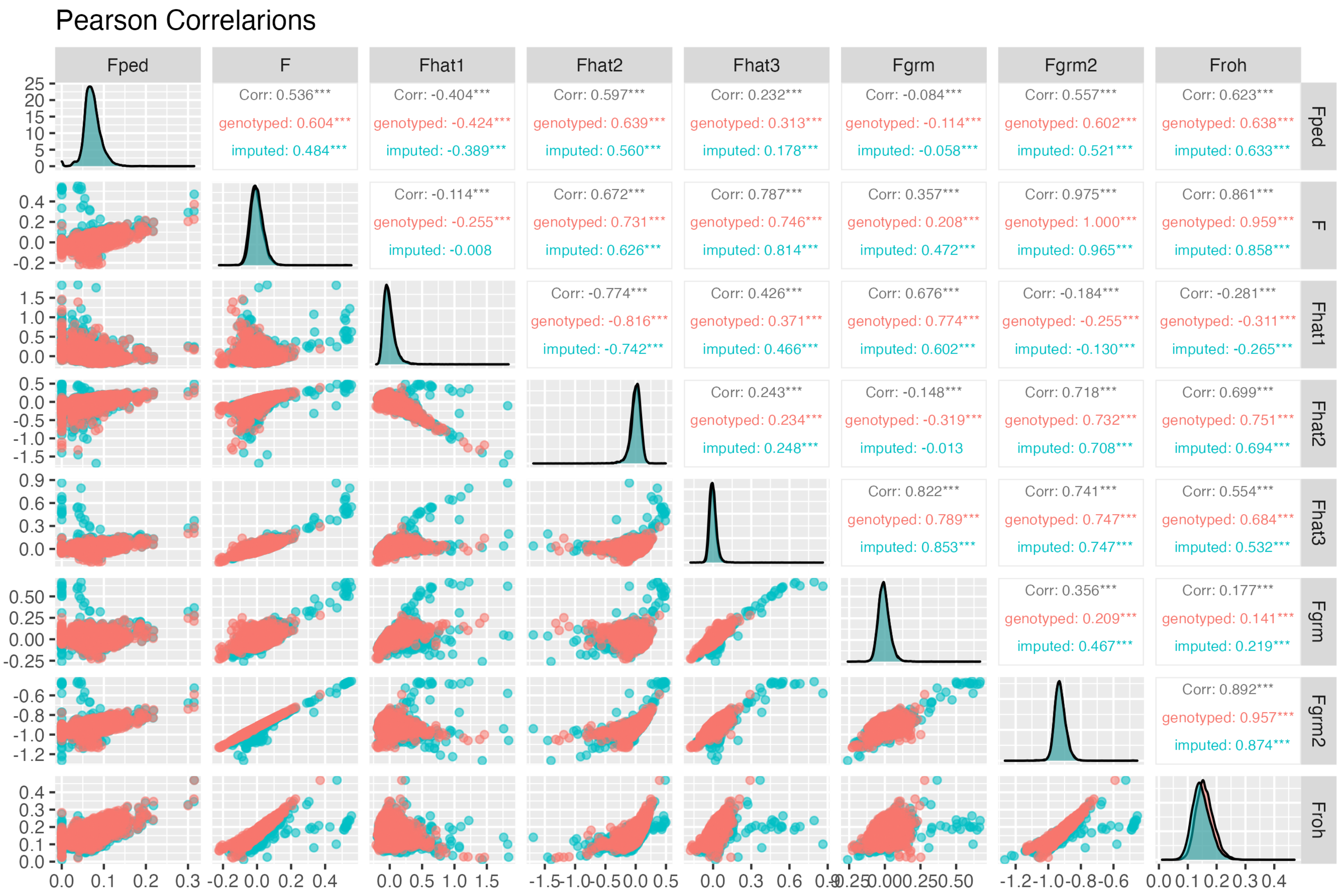

f) Labogena MD
